# Sampled-data exponential stabilization for delayed complex valued neural networks with quantized intermittent communication

**DOI:** 10.1371/journal.pone.0357795

**Published:** 2026-09-09

**Authors:** Jie Li, Xiaohong Wang

**Affiliations:** College of Science, Qingdao University of Technology, Qingdao, Shandong, China; University of Shanghai for Science and Technology, CHINA

## Abstract

This paper addresses the sampled-data exponential stabilization problem for delayed complex-valued neural networks (DCNNs) over capacity-limited communication channels. To reduce communication load, a quantized intermittent sampled-data transmission (QIST) protocol is proposed. A transmission-interval-dependent Lyapunov functional is constructed, which is continuous and piecewise-defined in accordance with the communication mechanism. Using the Lyapunov functional method combined with inequality estimation techniques, two exponential stabilization criteria are derived in the form of complex-valued linear matrix inequalities (LMIs). These criteria establish, for the first time, an explicit quantitative relationship between the duty cycle of the rest intervals and the maximum allowable upper bound of the transmission length. Specifically, it is shown that the maximum allowable duty cycle $\overset{―}{\Psi }$ decreases monotonically as the transmission length increases, and a one-dimensional search algorithm is provided to determine either $\overset{―}{\Psi }$ or the transmission length $h_{k}$. Numerical simulations on a third-order DCNN demonstrate that the proposed QIST protocol achieves global exponential stabilization with reduced communication resource consumption, and the trade-off between actuator rest time and switching frequency of electronic devices is quantitatively analyzed. These results confirm the effectiveness and practical advantages of the proposed approach.

## 1. Introduction

Study of delayed complex valued neural networks (DCNNs) has gained considerable attention because of their numerous uses in a variety of domains, including as control systems, image recognition, signal processing, and others [1–9]. DCNNs are a type of neural network that processes information in the complex field using complex parameters and variables. They have complex-valued state variables, activation functions, connection weight matrices, and external inputs [10–12]. Compared to traditional real valued neural networks, DCNNs perform information processing in the complex domain, which enables them to more accurately describe certain practical problems with complex properties. DCNNs are known to exhibit complex dynamical behaviors, and ensuring their stability is crucial for reliable operation.

The issue of stability is directly tied to stabilization. In many practical applications, stabilization is often used to achieve satisfactory results, such as fast convergence and required system performance. The stabilization of DCNNs has drawn even increasing attention and many interesting and remarkable results have been obtained [13–16]. Furthermore, some classical control methods, such as state-feedback control [17] and adaptive control [18]. However, these control methods are point-to-point, which is important to note. That is to say, the controller must receive real-time data information. That can result in wastage of power resources. As computer technology and wireless networks have advanced, the communication industry has become more concerned, and several networked resource-saving control approaches, such as sampled-data control [19–23], event-triggered control [24–28] have been developed. Thus, it is extremely significant to realize the stabilization of the DCNNs via the networked control.

Notice that the shared characteristic of networked control techniques is only updates the transmitted signals at some appointed instants. Sampled-data control in which only the sampled measures can be used, is easily implemented by the electronic devices. It can reduce the power consumption and costs while improving reliability. Accordingly, sampled-data control of neural networks have been well discussed. For example, [29] considers the mean-square exponential stability issue of memristive neural networks via the sampled-data control. The space-time sampled-data control issue for memristor-based reaction-diffusion neural networks has been developed [30]. It can not only achieve the desired system performance, but also significantly reduce network communication resource consumption by designing a space-time sampled-data control protocol. [31] investigates the exponential synchronization problem of a kind of delayed fuzzy memristive inertial neural networks with the help of the nonfragile memory sampled-data control.

However, in some cases, in order to save power or maintain equipment for control system, controllers or actuators may suspend purposefully their work, resulting in limited “transmission time” and sampled-data control can only operate intermittently. It gives rise to the intermittent control. It is natural to use the intermittent sampled-data transmission (IST) protocol when network communication is limited. Furthermore, [32] investigates the exponential synchronization for chaotic delayed neural networks by designing an IST scheme. [33] considers the local stability of neural networks subject to actuator saturation by designing an IST scheme. On the other hand, quantization is an efficient to keep communication lines open because the bandwidth and bits rate of communication channels are limited. Obviously, quantized intermittent sampled-data transmission (QIST) can further enhance communication efficiency and conserve limited communication resources. In spite of there are several results concerning IST [32–39] and quantized sampled-data transmission (QST) [40–45], quantized intermittent transmission (QIT) [46–48], few authors applied the QIST scheme to neural networks, let alone to DCNNs. Therefore, that motivates this paper.

This paper deals with the exponential stabilization of a kind of DCNNs via QIST scheme. Due to the activation of designed sampled-data controller only in the working time, the resulting controlled DCNNs are essentially a switched one between two modes. Such situation lead to the following issues: how to construct the Lyapunov functionals to correspond to the designed communication mechanism? how to relax the requirement of Lyapunov matrices while ensuring the continuity of the designed Lyapunov functional at switching instants? These issues have not been tackled before to our knowledge, which also motivates our this paper.

Based on above discussion, this paper investigates the exponential stabilization for a kind of DCNNs via a QIST scheme. The contributions of this paper are threefold:

(a) Different from existing quantized intermittent sampled-data transmission (QIST) schemes that are only developed for real-valued neural networks, this work establishes a complete QIST control framework for delayed complex-valued neural networks (DCNNs). A complex-domain state feedback controller is designed, which significantly cuts network transmission frequency and actuator running time to improve energy efficiency under limited communication bandwidth.
(b) A novel transmission-interval-dependent switched Lyapunov functional is specially constructed to match the two-mode switching characteristic of the QIST protocol (transmission interval $\left[t_{k},s_{k}\right)$ and rest interval $\left[s_{k},t_{k+1}\right)$). Compared with conventional Lyapunov-Krasovskii functionals for intermittent control, the proposed functional maintains continuity at switching instants and imposes much milder constraints on Lyapunov matrices, which fully exploits sampled state information to reduce conservatism.
(c) Combining sector-bounded quantization characterization and advanced integral inequality techniques, two less-conservative exponential stabilization criteria are derived in the form of complex-valued LMIs. For the first time, our conditions quantitatively reveal the explicit coupling relationship among exponential convergence rate, quantization density, maximum transmission length and rest-interval duty cycle. A one-dimensional search algorithm is further provided to solve the admissible upper bound of the duty cycle.

The explanations for some notations used in this paper are provided in Table 1. This paper is organized by five parts. A QIST scheme is given and the considered model description is presented and some necessary preparatory works are shown in Section 2. In Section 3, two exponential stabilization criteria are established and formulated as LMIs. In Section 4, a numerical example is presented to shown the improvement of the main theoretical results. Finally, this paper is concluded in Section 5 and some supporting information is shown in Section 6.

**Table 1:** List of notations.

Notations	Explanations
${\mathbb{R}}^{n}({\mathbb{C}}^{n})$	*n*-dimensional real (complex) Euclidean space
${\mathbb{R}}^{n\times n}({\mathbb{C}}^{n\times n})$	set of n×n real (complex) matrices
$M^{T}$	transpose of real matrix *M*
$M^{*}$	conjugation transpose of complex matrix *M*
*M* ^-1^	inverse of real square matrix *M*
diag{·}	n-dimensional diagonal matrix
$C\left(\left[m,n\right],{\mathbb{C}}^{n}\right)$	continuous function ϕ from [m,n] to ${\mathbb{C}}^{n}$ with the uniform norm $\left||\phi ||=\sup_{m\leq s\leq n}||\phi (s)|\right|$
Sym(Y)	$Y+Y^{T}$ or $Y+Y^{*}$
*A* > 0(<0)	the symmetric positive (negative) definite matrix
$\lambda_{\min}(P)(\lambda_{\max}(P))$	minimum (maximum) eigenvalue of the matrix *P*
$||s||=\sqrt{s^{*}s}$	modulus of $s\in {\mathbb{C}}^{n}$
col{·}	column vector
*	symmetric element in a symmetric matrix
$I_{n}$	*n*th order identity matrix
*N* ^+^	{1,2,⋯,n}
***i***	imaginary unit, $i=\sqrt{-1}$
*x*(*t*)=*Re*(*z*(*t*))	real part of *z*(*t*)
*y*(*t*)=*Im*(*z*(*t*))	imaginary part of *z*(*t*)

## 2. Problem formulation and Preliminaries

### 2.1. Model description

A class of DCNNs is considered as follows:


$$\begin{array}{c} \left\{ \begin{array}{c} \dot{z}(t)=-Dz(t)+Af(z(t))+Bg(z(t_{\tau }))+u(z(t)),\;t>0,\\ z(t)=\phi (s),\;t\in [-\tau ,0], \end{array}\right. \end{array}$$ (1)

where $z(t)=col\{z_{1}(t),z_{2}(t),\cdots ,z_{n}(t)\}\in C^{n}$ and $z_{i}(t)$ means the state vector of the *i*th node; $D=diag\{d_{1},d_{2},\cdots ,d_{n}\}$ denotes a n×n diagonal matrix with $d_{i}>0$, $i\in N^{+}$; $A=(a_{ij}{)}_{n\times n}$, $B=(b_{ij}{)}_{n\times n}\in C^{n\times n}$ represent the connection strength matrices and delayed connection strength matrices, respectively; $z(t_{\tau })=z(t-\tau (t))=col\{z_{1}(t_{\tau }),z_{2}(t_{\tau }),\cdots ,z_{n}(t_{\tau })\}\in C^{n}$ is the delay and τ(t) satisfies $0\leq \tau (t)\leq \tau_{M}$ and $\dot{\tau }(t)<a<1$; *f*(*z*(*t*)), $g(z(t_{\tau }))$ are nonlinear neural activation functions; $\phi (s)\in C([-\tau ,0],C^{n})$ is initial values of neural networks; *u*(*z*(*t*)) is the control input.

In this paper, for $i\in N^{+},$ nonlinear neural activation functions are subject to the following assumptions.

**Assumption 1.** *Nonlinear functions* $f_{i}(\cdot )$ *and* $g_{i}(\cdot )$ *are Lipschitz with the Lipschitz constants* $s_{i},\;l_{i}>0$ *such that for any* $u_{1},u_{2}\in \mathbb{C}$


$$\begin{array}{c} ||f_{i}(u_{1})-f_{i}(u_{2})||\leq s_{i}||u_{1}-u_{2}||, \end{array}$$

$$\begin{array}{c} ||g_{i}(u_{1})-g_{i}(u_{2})||\leq l_{i}||u_{1}-u_{2}||. \end{array}$$

**Assumption 2.** *The component functions* $f_{i}(\cdot )$ *and* $g_{i}(\cdot )$ *satisfy*


$$\begin{array}{c} f_{i}(0)=g_{i}(0)=0. \end{array}$$

**Definition 1.** [52] *System* (1) *is globally exponentially stabilized, if there exist positive constants* ϱ *and M, such that*


$$\begin{array}{c} ||z(t)||\leq M||z_{0}||e^{-\varrho t},t\geq 0. \end{array}$$

**Lemma 1.** [49] *The Hermitian matrix Q is negative definite if*


$$\begin{array}{c} \left[\begin{array}{cc} Q_{1} & -Q_{2}\\ Q_{2} & Q_{1} \end{array}\right]<0, \end{array}$$

*where Q*_*1*_ *=Re(Q) and Q*_*2*_ *=Im(Q).*

### 2.2. The QIST protocol

**Fig 1:**
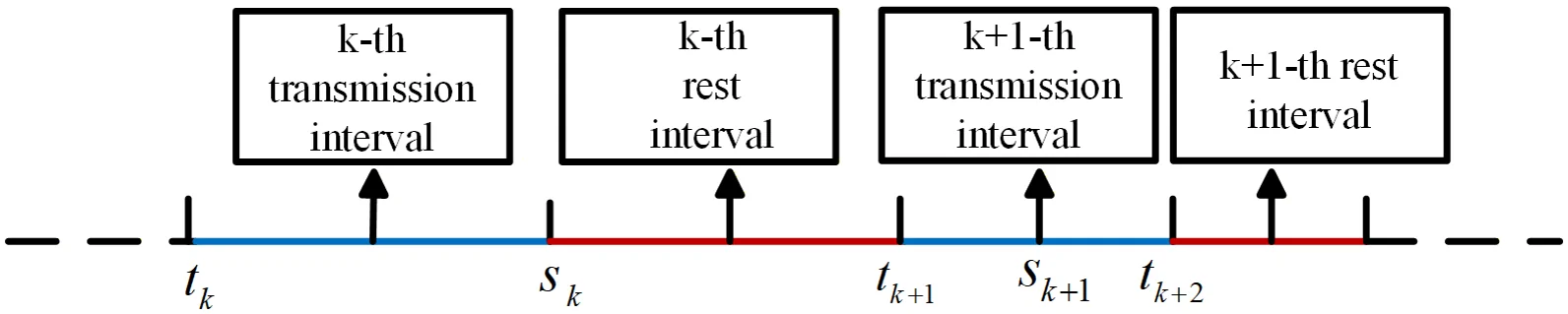
Time diagram of the QIST protocol (2).

This paper considers the exponential stabilization of system (1) under the framework of the networked system. The controlled system (1), sensors, quantizers, controllers, the zero-order hold (ZOH), actuators, etc., are all connected together through digital communication channels. Considering the control cost and limited network resources brought by actuators, redundant signal transmission may lead to heavy transmission burden. Therefore, a quantized intermittent sampling transmission mechanism is designed. A switching function *S*(*t*) is set between the actuator and ZOH, and it is 1 when system (1) in the transmission intervals, i.e., $t\in [t_{k},s_{k})$; it is 0 when system (1) in the rest intervals, i.e., $t\in [s_{k},t_{k+1})$. The sampled-data is quantized by a static quantizer and then transmitted to the controller. The QIST protocol is shown in Fig 1. Therefore, the controller designed is as follows:


$$\begin{array}{c} u(t)=\left\{ \begin{array}{c} Kq(z(t_{k})),S(t)=1,t\in [t_{k},s_{k})\\ 0,S(t)=0,t\in [s_{k},t_{k+1}) \end{array}\right. \end{array}$$ (2)

where *K* is the designed feedback gains, $q(z(t_{k}))=col\{q_{1}(z(t_{k})),q_{2}(z(t_{k})),\cdots ,q_{n}(z(t_{k}))\}$, which is symmetric, static and time-invariant. The quantizer is defined as


$$\begin{array}{c} q(v)=\left\{ \begin{array}{c} u_{i},if\;\frac{u_{i}}{1+\delta }<v<\frac{u_{i}}{1+\delta }\;and\;v>0,\\ 0,if\;v=0,\\ -q(v),if\;v<0, \end{array}\right. \end{array}$$ (3)

where $\delta =\frac{1-\rho }{1+\rho }$. $V=\{\pm u_{i}:u_{i}=\rho^{i}u_{0},i\in N^{+}\}\cup \{\pm u_{0}\}\cup \{0\}$ is the set of quantized levels; 0<ρ<1 is the parameter about quantization density; positive parameter *u*_0_ is the initial value of this quantizer. The logarithmic quantizer in (3) is adopted for three main reasons. First, it admits a convenient sector-bound description q(v)−v=Δ(t)v with ||Δ(t)||≤δ, which enables the quantization error to be incorporated into the subsequent LMI-based stability analysis in a tractable manner. Second, the quantization density parameter ρ∈(0,1) provides a direct trade-off between quantization accuracy and communication bit rate, which is crucial for capacity-limited channels. Third, this type of quantizer has been widely validated in [40]–[45] and is standard for neural network systems with quantized feedback. It is worth noting that other quantizer types, such as uniform quantizers, dynamic quantizers with adjustable scaling, or hysteresis quantizers, could also be considered in principle. However, uniform quantizers generally lack the sector-bound property that facilitates LMI-based design, while dynamic and hysteresis quantizers would introduce additional dynamics or switching logic that complicate the stability analysis. Extending the proposed framework to accommodate these alternative quantization schemes is an interesting direction for future investigation. $\left[t_{k},t_{k+1}\right)$ is the control length, $h_{k}=s_{k}-t_{k}$ is the transmission length, $h_{k}\in [h_{1},h_{2}]$. Then, one expresses system (1) with controller (2) as


$$\begin{array}{c} \dot{z}(t)=-Dz(t)+Af(z(t))+Bg(z(t_{\tau }))+S(t)Kq(z(t_{k})),t\in [t_{k},t_{k+1}). \end{array}$$ (4)

By using the sector bound method proposed in [50], one can express the quantization error as follows:


$$\begin{array}{c} q(z(t_{k}))-z(t_{k})=\Delta (t_{k})z(t_{k}), \end{array}$$ (5)

where $||\Delta (t_{k})||\leq \delta .$

When $t\in [t_{k},t_{k+1})$, substituting(5) into (4), one gets the following controlled system


$$\begin{array}{c} \dot{z}(t)=-Dz(t)+Af(z(t))+Bg(z(t_{\tau }))+S(t)K(I+\Delta (t_{k}))q(z(t_{k})). \end{array}$$ (6)

The following definitions are shown for the QIST protocol (2) to analyze the stability and the performance of DCNNs (6).

**Definition 2.** *Duty cycle of the rest interval for the protocol (2) is defined as:*


$$\begin{array}{c} \Psi =\lim_{k\to +\infty }\sup\frac{t_{k+1}-s_{k}}{t_{k+1}-t_{k}}. \end{array}$$

**Definition 3.** [51] *For any* $k\in N^{+}\cup \{0\}$*, if one has*


$$\begin{array}{c} \Psi (t)=\frac{t-s_{k}}{t-t_{k}},t\in [s_{k},t_{k+1}]. \end{array}$$

*then, it increases monotonically, this is,* $\Psi (t)\leq \Psi (t_{k+1})$.

**Remark 1.** *In this paper, taking the state quantization into consideration, a QIST protocol is given to study the exponential stability issue of DCNNs with the aim of overcoming the limited communication capabilities and improving the efficiency of DCNNs. Time interval* $\left[t_{k},t_{k+1}\right)$ *between two sequentially sampled events is segmented into two parts by designed QIST protocol (2):* $\left[t_{k},s_{k}\right)$ *and* $\left[s_{k},t_{k+1}\right)$*. Wherein* $\left[t_{k},s_{k}\right)$ *means the time when the sampled-data transmitted over the communication channel;* $\left[s_{k},t_{k+1}\right)$ *denotes the time when the communication channel does not work.* $h_{k}=0$ *stands for there is no sampled-data transmission. Therefore, the designed QIST protocol (2) has more advantages over the protocols in [32]–[48] in saving both network resources and control cost and reducing effectively both the information transmission amount and channel blocking.*

## 3. Stability analysis

This section is dedicated to investigating the globally exponential stability of controlled networks (6). Two sufficient conditions are derived as follows.

**Theorem 1.** *Under Assumption 1 and Assumption 2, for given positive scalars* $h_{2}\geq h_{1},\;\sigma ,\;\rho ,\;\alpha ,\;\Psi \in [0,1),$ *and real feedback gains K*, controlled system (6) ca*n achieve exponential stabilization, if there exist* n×n *real symmetric matrices P > 0, R > 0, Q > 0,* n×n *real diagonal matrices* $\Lambda_{1}>0,\;\Lambda_{2}>0$ *and* n×n *real matrices U, M, W and N as free-weighting matrices, such that for any* $h_{k}\in \{h_{1},h_{2}\}$*, the following LMIs hold*


$$\begin{array}{c} \Pi (h_{k})=\left[\begin{array}{cc} P+h_{k}Sym(\frac{X}{2}) & h_{k}(-X+Y)\\ {}* & h_{k}Sym(-Y+\frac{X}{2}) \end{array}\right]>0, \end{array}$$ (7)

$$\begin{array}{c} F_{0}(h_{k})=\left[\begin{array}{cc} {}[\gamma_{ij}{]}_{6n\times 6n} & [\Pi_{ij}{]}_{6n\times 2n}\\ {}* & -I_{2n} \end{array}\right]<0, \end{array}$$ (8)

$$\begin{array}{c} F_{1}(h_{k})=\left[\begin{array}{cc} {}[\Delta_{ij}{]}_{7n\times 7n} & [\theta_{ij}{]}_{7n\times 2n}\\ {}* & -I_{2n} \end{array}\right]<0, \end{array}$$ (9)

$$\begin{array}{c} F_{2}=\left[\begin{array}{cc} {}[\Phi_{ij}{]}_{3n\times 3n} & [\Gamma_{ij}{]}_{3n\times 2n}\\ {}* & [\Lambda_{ij}{]}_{2n\times 2n} \end{array}\right]<0, \end{array}$$ (10)

$$\begin{array}{c} \varrho =2[\sigma (1-\Psi )-\alpha \Psi ]>0, \end{array}$$ (11)

*where*


$$\begin{array}{cc} \gamma_{11} & =2\sigma P+Q+(2\sigma h_{k}-1)Sym\left(\frac{X}{2}\right)+F\Lambda_{1}F-Sym(MD)+I,\\ \gamma_{12} & =P+h_{k}Sym\left(\frac{X}{2}\right)-M-DU^{T},\\ \gamma_{14} & =(2\sigma h_{k}-1)(Y-X)+MK,\\ \gamma_{15} & =\Delta_{15}=MA,\;\gamma_{16}=\Delta_{16}=MB,\\ \gamma_{22} & =h_{k}R+I-Sym(U),\;\gamma_{24}=UK+h_{k}(Y-X),\\ \gamma_{25} & =\Delta_{25}=UA,\;\gamma_{26}=\Delta_{26}=UB,\\ \gamma_{33} & =\Delta_{33}=\Phi_{33}=(a-1)\exp(-2\sigma \tau_{M})Q+G\Lambda_{2}G,\\ \gamma_{44} & =(2\sigma h_{k}-1)Sym\left(\frac{X}{2}-Y\right),\\ \gamma_{55} & =\Delta_{55}=\Lambda_{11}=-\Lambda_{1},\\ \gamma_{66} & =\Delta_{66}=\Lambda_{22}=-\Lambda_{2},\\ \Pi_{41} & =\theta_{41}=\delta UK,\\ \Pi_{42} & =\theta_{42}=\delta MK,\\ \Delta_{11} & =2\sigma P+Q-Sym\left(\frac{X}{2}+MD\right)+F\Lambda_{1}F+I,\\ \Delta_{12} & =P-M-DU^{T},\;\Delta_{14}=X-Y+MK,\\ \Delta_{22} & =I-Sym(U),\\ \Delta_{44} & =Sym\left(Y-\frac{X}{2}\right),\\ \Delta_{77} & =-h_{k}\exp(-2\sigma h_{2})R,\\ \Phi_{11} & =-2\alpha P+Q-Sym(ND)+F\Lambda_{1}F,\\ \Phi_{12} & =P-N-DW^{T},\\ \Phi_{22} & =-Sym(W),\\ \Gamma_{11} & =NA,\;\Gamma_{12}=NB,\\ \Gamma_{21} & =WA,\;\Gamma_{22}=WB. \end{array}$$

**Proof:** One considers piecewise Lyapunov function as following


$$\begin{array}{c} V(t)=\left\{ \begin{array}{c} \sum_{i=1}^{4}V_{i}(t),\;t\in [t_{k},s_{k})\\ \sum_{i=1}^{2}V_{i}(t),\;t\in [s_{k},t_{k+1}) \end{array}\right. \end{array}$$

where


$$\begin{array}{c} V_{1}(t)=z^{*}(t)Pz(t), \end{array}$$

$$\begin{array}{c} V_{2}(t)=\int_{t-\tau (t)}^{t}e^{2\sigma (s-t)}z^{*}(s)Qz(s)ds, \end{array}$$

$$\begin{array}{c} V_{3}(t)=(s_{k}-t)\int_{t_{k}}^{t}e^{2\sigma (s-t)}{\dot{z}}^{*}(s)R\dot{z}(s)ds, \end{array}$$

$$\begin{array}{c} V_{4}(t)=(s_{k}-t)\xi^{*}(t)H\xi (t), \end{array}$$

and


$$\begin{array}{c} \xi (t)=col\{z(t),z(t_{k})\}, \end{array}$$

$$\begin{array}{c} \begin{array}{c} H=\left[\begin{array}{cc} Sym(\frac{X}{2}) & -X+Y\\ {}* & Sym(-Y+\frac{X}{2}) \end{array}\right]. \end{array} \end{array}$$

Take the derivative of *V*(*t*) along the trajectories (6) yields


$$\begin{array}{c} {\dot{V}}_{1}(t)+2\sigma V_{1}(t)=Sym\{z^{*}(t)P\dot{z}(t)\}+2\sigma z^{*}(t)Pz(t), \end{array}$$ (12)

$$\begin{array}{cc} {\dot{V}}_{2}(t)+2\sigma V_{2}(t) & \leq z^{*}(t)Qz(t)-e^{-2\sigma \tau_{M}}(1-a)z^{*}(t_{\tau })Qz(t_{\tau }), \end{array}$$ (13)

$$\begin{array}{c} {\dot{V}}_{3}(t)+2\sigma V_{3}(t)=-\int_{t_{k}}^{t}e^{2\sigma (s-t)}{\dot{z}}^{*}(s)R\dot{z}(s)ds+(s_{k}-t){\dot{z}}^{*}(t)R\dot{z}(t), \end{array}$$ (14)

$$\begin{array}{cc} {\dot{V}}_{4}(t)+2\sigma V_{4}(t) & =[2\sigma (s_{k}-t)-1]\xi^{*}(t)H\xi (t)+(s_{k}-t)Sym[{\dot{z}}^{*}(t)Sym(\frac{X}{2})z(t)+{\dot{z}}^{*}(t)(-X+Y)z(t_{k})]. \end{array}$$ (15)

According to the inequality in [52], we obtain


$$\begin{array}{c} (t-t_{k})v^{*}(t)Rv(t)\leq \int_{t_{k}}^{t}{\dot{z}}^{*}(s)R\dot{z}(s)ds, \end{array}$$

where $v(t)=\frac{\int_{t_{k}}^{t}\dot{z}(s)ds}{t-t_{k}}.$ Thus, equation (14) becomes


$$\begin{array}{c} {\dot{V}}_{3}(t)+2\sigma V_{3}(t)\leq -e^{-2\sigma h_{2}}(t-t_{k})v^{*}(t)Rv(t)+(s_{k}-t){\dot{z}}^{*}(t)R\dot{z}(t). \end{array}$$ (16)

With Assumption 2, for diagonal matrices $\Lambda_{1}>0,\;\Lambda_{2}>0$, one has:


$$\begin{array}{c} f^{*}(z(t))\Lambda_{1}f(z(t))-z^{*}(t)F\Lambda_{1}Fz(t)\leq 0,\\ g^{*}(z(t_{\tau }))\Lambda_{2}g(z(t_{\tau }))-z^{*}(t_{\tau })G\Lambda_{2}Gz(t_{\tau })\leq 0. \end{array}$$ (17)

When $t\in [t_{k},s_{k})$, for auxiliary matrices $U,M\in R^{n\times n}$, the following equalities hold


$$\begin{array}{cc} 0 & =Sym([z^{*}(t)M+{\dot{z}}^{*}(t)U]\times [-Dz(t)+Af(z(t))+Bg(z(t_{\tau }))+K(I+\Delta (t_{k}))z(t_{k})-\dot{z}(t)])\\ & \leq Sym[-z^{*}(t)MDz(t)+z^{*}(t)MAf(z(t))+z^{*}(t)MBg(z(t_{\tau }))+z^{*}(t)MKz(t_{k})-z^{*}(t)M\dot{z}(t)\\ & \;-{\dot{z}}^{*}(t)UDz(t)+{\dot{z}}^{*}(t)UAf(z(t))+{\dot{z}}^{*}(t)UBg(z(t_{\tau }))+{\dot{z}}^{*}(t)UKz(t_{k})-{\dot{z}}^{*}(t)U\dot{z}(t)]\\ & \;+z^{*}(t)z(t)+z^{*}(t_{k})\delta^{2}K^{T}M^{T}MKz(t_{k})+z^{*}(t_{k})\delta^{2}K^{T}U^{T}UKz(t_{k})+{\dot{z}}^{*}(t)\dot{z}(t). \end{array}$$ (18)

According to (12–13) and (15)–(18), we have


$$\begin{array}{c} \dot{V}(t)+2\sigma V(t)\leq \frac{s_{k}-t}{h_{k}}{\eta_{0}}^{*}(t)F_{0}(h_{k})\eta_{0}(t)+\frac{t-t_{k}}{h_{k}}\eta^{*}(t)F_{1}(h_{k})\eta (t),h_{k}\in \{h_{1},h_{2}\} \end{array}$$

and $\eta (t)=col\{\eta_{0}(t),v(t)\},\eta_{0}(t)=col\{z(t),\dot{z}(t),z(t_{\tau }),z(t_{k}),f(z(t)),g(z(t_{\tau }))\},F_{0}(h_{k}),F_{1}(h_{k})$ have been defined in Theorem 1.

Then, inequalities (8) and (9) imply


$$\begin{array}{c} \dot{V}(t)+2\sigma V(t)<0,\;\forall \;\eta (t)\neq 0. \end{array}$$

Thus, one has


$$\begin{array}{c} V(t)<V(t_{k})e^{-2\sigma (t-t_{k})}. \end{array}$$

When $t\in [s_{k},t_{k+1})$, for auxiliary matrices $W,N\in R^{n\times n}$, one has


$$\begin{array}{c} 0=Sym[{\dot{z}}^{*}(t)W+z^{*}(t)N]\times [-Dz(t)+Af(z(t))+Bg(z(t_{\tau }))-\dot{z}(t)]. \end{array}$$ (19)

Based on (12)–(13), (17) and (19), one can obtain


$$\begin{array}{c} \dot{V}(t)-2\alpha V(t)\leq \eta_{1}^{*}(t)F_{2}\eta_{1}(t), \end{array}$$

where $\eta_{1}(t)=col\{z(t),\dot{z}(t),z(t_{\tau }),f(z(t)),g(z(t_{\tau }))\}$.

Inequality (10) implies


$$\begin{array}{c} \dot{V}(t)-2\alpha V(t)<0. \end{array}$$

Then, one can also acquire


$$\begin{array}{c} V(t)<V(s_{k})e^{2\alpha (t-s_{k})}. \end{array}$$

Based on the definition of *V*(*t*), it is clear that


$$\begin{array}{c} V_{3}(t_{k})=\lim_{t\to t_{k}^{-}}V_{3}(t_{k})=0,V_{4}(t_{k})=\lim_{t\to t_{k}^{-}}V_{4}(t_{k})=0, \end{array}$$

$$\begin{array}{c} V_{3}(s_{k})=\lim_{t\to s_{k}^{-}}V_{3}(s_{k})=0,V_{4}(s_{k})=\lim_{t\to s_{k}^{-}}V_{4}(s_{k})=0. \end{array}$$

Then, we obtain


$$\begin{array}{c} \lim_{t\to s_{k}^{-}}V(t)=V(s_{k}),\lim_{t\to t_{k}^{-}}V(t)=V(t_{k}). \end{array}$$

Thus, *V*(*t*) is continuous when $t=t_{k}$ and $t=s_{k}$.

Additionally, for $t\in [t_{k},s_{k})$, we have


$$\begin{array}{c} \begin{array}{cc} V(t) & \leq e^{-2\sigma (t-t_{k})}V(t_{k})\\ & \leq e^{2\alpha (t_{k}-s_{k-1})}e^{-2\sigma (t-t_{k})}V(s_{k-1})\\ & \leq e^{-2\sigma (s_{k-1}-t_{k-1})}e^{2\alpha (t_{k}-s_{k-1})}e^{-2\sigma (t-t_{k})}V(t_{k-1})\\ & \leq ...\\ & \leq e^{-2\sigma \sum_{r=0}^{k-1}(s_{r}-t_{r})+2\alpha \sum_{r=0}^{k-1}(t_{r+1}-s_{r})}e^{-2\sigma (t-t_{k})}V(0)\\ & =e^{-2\sigma \sum_{r=0}^{k-1}(1-\frac{t_{r+1}-s_{r}}{t_{r+1}-t_{r}})(t_{r+1}-t_{r})+2\alpha \sum_{r=0}^{k-1}\frac{t_{r+1}-s_{r}}{t_{r+1}-t_{r}}(t_{r+1}-t_{r})}e^{-2\sigma (t-t_{k})}V(0)\\ & \leq e^{-2\sigma (1-\Psi )t_{k}+2\alpha \Psi t_{k}}e^{-2\sigma (t-t_{k})}V(0)\\ & =e^{2\Psi (\sigma +\alpha )t_{k}}e^{-2\sigma t}V(0)\\ & \leq e^{2\Psi (\sigma +\alpha )t}e^{-2\sigma t}V(0)\\ & =e^{-2[\sigma (1-\Psi )-\alpha \Psi ]t}V(0)\\ & =e^{-\varrho t}V(0). \end{array} \end{array}$$

For $t\in [s_{k},t_{k+1})$, one also obtains


$$\begin{array}{c} V(t)\leq e^{-\varrho t}V(0). \end{array}$$ (20)

Besides, we can have


$$\begin{array}{c} V_{1}(t)+V_{4}(t)=\xi^{*}(t)[\frac{s_{k}-t}{h_{k}}\Pi (h_{k})+\frac{t-t_{k}}{h_{k}}\Pi (0)]\xi (t)\leq V(t). \end{array}$$

According to (7) and *P*,*Q* > 0, there exist n-order and 2n-order identity matrices $I_{n}$,*I*_2*n*_, and sufficiently small positive scalar ϵ, such that


$$\begin{array}{c} P>\epsilon I_{n},Q>\epsilon I_{n},\Pi (h_{k})>\epsilon I_{2n}. \end{array}$$

Thus, one has


$$\begin{array}{c} \epsilon ||z(t)|{|}^{2}\leq V(t). \end{array}$$

In addition, one can see


$$\begin{array}{cc} V(0) & =z^{*}(0)Pz(0)+\int_{-\tau_{M}}^{0}e^{2\sigma s}z^{*}(s)Qz(s)ds\\ & \leq z^{*}(0)Pz(0)+\int_{-\tau_{M}}^{0}z^{*}(s)Qz(s)ds\\ & \leq \lambda_{\max}(P)||z(0)|{|}^{2}+\tau_{M}\lambda_{max}(Q)\underset{-\tau_{M}\leq \zeta \leq 0}{\sup}||z(\zeta )|{|}^{2}\\ & \leq \theta_{1}(\underset{-\tau_{M}\leq \zeta \leq 0}{\sup}\{||z(\zeta )|{|}^{2},||\dot{z}(\zeta )|{|}^{2}\}), \end{array}$$ (21)

where $\theta_{1}=\lambda_{\max}(P)+\tau_{M}\lambda_{\max}(Q)$.

One obtains from (20)–(21) that


$$\begin{array}{c} ||z(t)||\leq \sqrt{\frac{\theta_{1}}{\epsilon }}e^{-\varrho t}||z_{0}||=\sqrt{\frac{\theta_{1}}{\epsilon }}e^{-\varrho t}\phi (s). \end{array}$$

Therefore,


$$\begin{array}{c} \lim_{t\to +\infty }V(t)=0, \end{array}$$

which showns that controlled DCNNs (6) is globally exponentially stabilized. The proof is completed.

**Remark 2.** *With the intention of reducing the conservativeness in the process of analyzing system stability, on one hand, the construction of a transmission-interval-dependent Lyapunov functional can make the best of the available measurement over the transmission intervals* $\left[t_{k},s_{k}\right)$*. It is also switched in two segmented intervals* $\left[t_{k},s_{k}\right)$ *and* $\left[s_{k},t_{k+1}\right)$ *which suits to characteristics of the QIST protocol (*2*). It is continuous and non-increasing which can ensure the smooth switching at the instants* $t_{k}$ *and* $s_{k}$*. On the other hand, some free-weighting matrices are introduced, which contribute to reducing the conservativeness of ensuring stability conditions. In brief, the stability criteria derived in Theorem 1 via the Lyapunov-based analysis method and inequality estimation techniques has less conservativeness.*

According to Theorem 1, one can now design the feedback gain matrix *K* to guarantee the neural networks (6) is exponentially stable.

**Theorem 2.** *Given parameters* $0<h_{1}\leq h_{2},\;\sigma ,\;\alpha ,\;\beta ,\;\Psi$*, system (6) can achieve exponential stabilization via control law (2), if the gain matrix is given by K = M*^*-1*^*V.*

**Proof:** Let U=βM,V=MK in (7)–(11). One can easily obtain the gain matrix via computing LMIs (7)-(11) with the help of MATLAB. The subsequent proof follows from Theorem 1.

**Remark 3.** *Theorem 2 gives co-design strategy for both the gain matrix and the transmission length* $h_{k}$*. Accordingly, one can not only obtain the desired control gain matrix via solving condtions derived in Theorem 2, but also can analyze the qualitative relationship between the transmission length* $h_{k}$ *and the duty cycle of the rest intervals. Therefore, it holds practical significance in engineering applications.*

**Remark 4.** *It is noted that the derived stability conditions involve LMIs with matrix dimensions up to* 9n×9n *(e.g.,* $F_{1}(h_{k})$ *in Theorem 1) and the number of scalar decision variables is of order* $𝒪(n^{2})$*. At first glance, this may raise concerns regarding computational burden. However, two facts should be considered. First, in most practical neural network applications, the number of neurons n is moderate (typically* n≤10 *or slightly higher), for which the LMIs can be solved extremely fast via MATLAB LMI Toolbox. Second, all LMIs are convex and strictly linear in the matrix variables, making them numerically tractable even for larger n on modern computing platforms. For instance, in the numerical example of Section* 4 w*ith n = 3, the feasible solution is obtained within milliseconds. For networks with up to dozens of neurons, the solution time remains acceptable for design purposes. Therefore, while the theoretical dimensionality is high, the practical implementation via standard LMI toolboxes does not compromise the applicability of the proposed method.*

In practical engineering applications, two main factors to be considered are actuator costs and the times of electronic devices updates. Generally, a larger Ψ indicates a shorter operating duration for the actuator, while a larger $h_{k}$ means a lower the frequency of updates for electronic devices. That is to say, we need to select the larger values of Ψ and $h_{k}$. Define $\overset{―}{\Psi }=\frac{\sigma }{\alpha +\sigma }$ is the maximum allowable value of Ψ(t) inspired the inequality (11). It is monotonically increasing about σ. It means a larger σ needs to be selected to obtain a larger $\overset{―}{\Psi }$. For simplicity, one supposes $h_{1}=h_{2}=h_{k}$. Then, Under the premise of maintaining performance of networks (6), a search algorithm for computing $\overset{―}{\Psi }$ or $h_{k}$ is devised as follows:

Step 1: Set α and $h_{k}$, select σ and iteration step size Δσ>0;

Step 2: Change σ=σ+Δσ;

Step 3: Computing the LMIs (7)–(11), if there is a feasible solution, return to execute Step 2. Otherwise, output $\overset{―}{\sigma }=\sigma -\Delta \sigma$;

Step 4: Fixed α and σ, the desired $h_{k}$ can be acquired via repeating Step 1–3.

**Remark 5.** *The proposed QIST-based stabilization method is primarily developed for delayed complex-valued neural networks (DCNNs) with moderate dimensions. Regarding its extensibility to large-scale interconnected systems, the LMI-based framework can, in principle, be extended by incorporating decentralized control architectures, where each subsystem is equipped with its own local QIST controller. However, for systems with hundreds of neurons, the centralized LMI approach may become computationally demanding; in such cases, decentralized or distributed implementations could be explored using sum-separable Lyapunov functions. It is worth noting that the problem setting in this paper differs from that of decentralized adaptive control for large-scale nonlinear systems with random couplings and biased measurements in* [53]*, as the latter involves adaptive mechanisms and output-feedback compensation, whereas our work focuses on state-feedback stabilization with quantization and intermittent communication. Extending the QIST framework to such decentralized adaptive settings for large-scale DCNNs remains an interesting direction for future research.*

**Remark 6.** *The parameters* $h_{k}$*,* Ψ*,* σ *and* α *are interrelated through the feasibility conditions of LMIs (7)–(11). Owing to the nonlinear coupling among these parameters and the system matrices, no closed-form analytical expression exists to describe the exact admissible region. Instead, the relationships are quantitative and must be determined numerically. Specifically, inequality (11) provides a necessary linear boundary* ρ=2[σ(1−Ψ)−αΨ]>0*, yet the actual feasible set is further constrained by the LMI conditions (7)–(10), which couple* $h_{k}$ *with the Lyapunov matrices. Consequently, the parameter boundaries in* Tables 2 and 3 *are not arbitrary trial results but are obtained via a systematic one-dimensional search algorithm (see Step 1–Step 4 in Sec*tion 3) *with prescribed resolution (e.g.,* $\Delta h_{k}=0.01$*,* $\Delta \overset{―}{\Psi }=10\%$*). This procedure is standard in LMI-based control design for sampled-data and intermittent systems and yields a practical, rigorous characterization of the admissible parameter margins. Hence, the presented numerical tables serve as a valid “quantitative relational characterization” of the parameter boundaries.*

**Table 2:** Qualitative relationship between *h*_2_ and $\overset{―}{\sigma }$, $\overset{―}{\Psi }$ in Theorem 1.

*h*_2_	0.01	0.02	0.03	0.04	0.05
$\overset{―}{\sigma }$	0.55	0.51	0.48	0.44	0.40
$\overset{―}{\Psi }$	57.9%	56%	54.5%	52.4%	50%

**Table 3:** Qualitative relationship between *h*_2_ and $\overset{―}{\Psi }$, $S_{\min}$ in Theorem 2.

$\overset{―}{\Psi }$	30%	40%	50%	60%	70%
*h*_2_	0.42	0.34	0.25	0.17	0.08
$S_{\min}$	59	73	100	147	312

## 4. Numerical simulation

**Fig 2:**
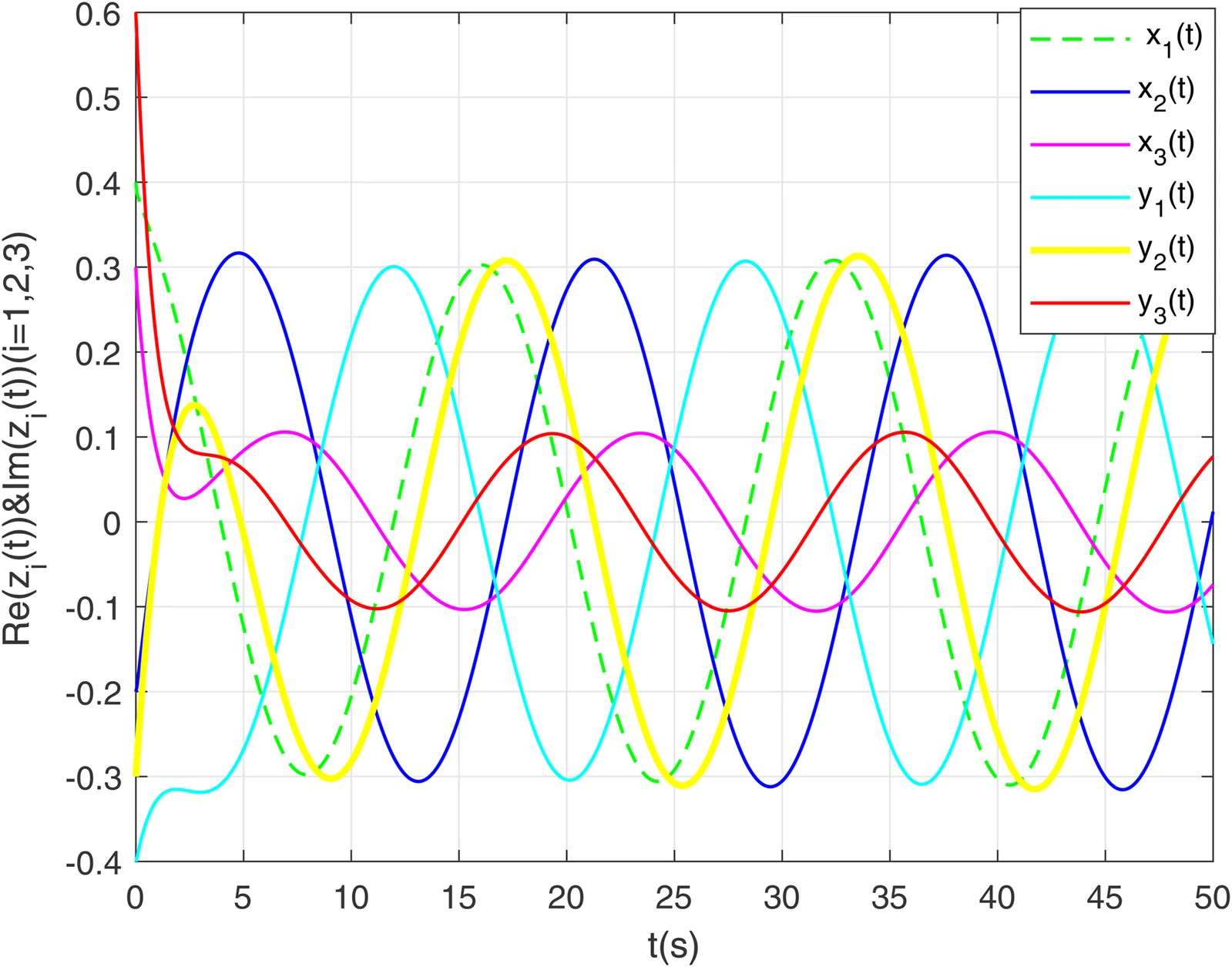
Time evolutions of real and imaginary parts for uncontrolled DCNNs (1).

Let us consider DCNNs whose parameters D=diag{0.8,0.8,0.8},


$$\begin{array}{c} A=\left[\begin{array}{ccc} 2.2-1.7i & -1-0.1i & -0.1+0.1i\\ -1.3+1.8i & 0.7-0.2i & -0.5+0.2i\\ -1.4-1.9i & 0.8+0.2i & -0.3-0.2i \end{array}\right], \end{array}$$

$$\begin{array}{c} B=\left[\begin{array}{ccc} 1.2+1.5i & -0.7+0.2i & -0.3+0.4i\\ 1.3+1.7i & 0.6-0.3i & -0.4+0.5i\\ 1.2+1.6i & 0.9+0.1i & -0.3+0.2i \end{array}\right], \end{array}$$

and nonlinear functions g(z(t))=f(z(t))=tanh(z(t)) are given as the same as [54]; the initial values $\phi (s)=[0.4-0.4i,-0.2-0.3i,0.3+0.6i{]}^{T}$, the time-varying delay $\tau (t)=\frac{e^{t}}{10(1+e^{t})}$. One can see τ(t) is bounded with $\tau_{M}=0.1$ and $\dot{\tau }(t)\leq 0.025<1$. The time evolutions of uncontrolled DCNNs (1) are depicted in Fig 2, which showns that the zero solution of the network is unstable.

In Theorem 1, set$h_{1}=h_{2}=h_{k}=0.02,\;\alpha =0.4,\;\sigma =0.5,\;\Psi =0.01$, feedback gain K=diag{−2,−2,−2}, the initial quantization *u*_0_ = 0.2, quantizer density ρ=0.5, then the quantization parameter is $\delta =\frac{1-\rho }{1+\rho }=\frac{1}{3}$. Based on Lemma 1, one solves (7)–(11) via LMI toolbox and obtain the following feasible solution:


$$\begin{array}{c} \varrho =2[\sigma (1-\Psi )-\alpha \Psi ]=0.982>0, \end{array}$$

$$\begin{array}{c} P=\left[\begin{array}{ccc} 4.4714 & 0.1542 & -0.1331\\ 0.1542 & 4.4449 & -0.3174\\ -0.1331 & -0.3174 & 3.3116 \end{array}\right], \end{array}$$ (22)

$$\begin{array}{c} Q=\left[\begin{array}{ccc} 1.7921 & 0.0376 & -0.0465\\ 0.0376 & 1.8786 & -0.1054\\ -0.0465 & -0.1054 & 1.0421 \end{array}\right], \end{array}$$ (23)

$$\begin{array}{c} R=\left[\begin{array}{ccc} 0.6114 & 0.5743 & -0.5039\\ 0.5743 & 3.7513 & -1.2892\\ -0.5039 & -1.2892 & 1.4219 \end{array}\right], \end{array}$$ (24)

$$\begin{array}{c} \Lambda_{1}=diag\{62.5461,44.7337,11.3376\}, \end{array}$$ (25)

$$\begin{array}{c} \Lambda_{2}=diag\{38.6648,36.7819,19.1839\}. \end{array}$$ (26)

**Fig 3:**
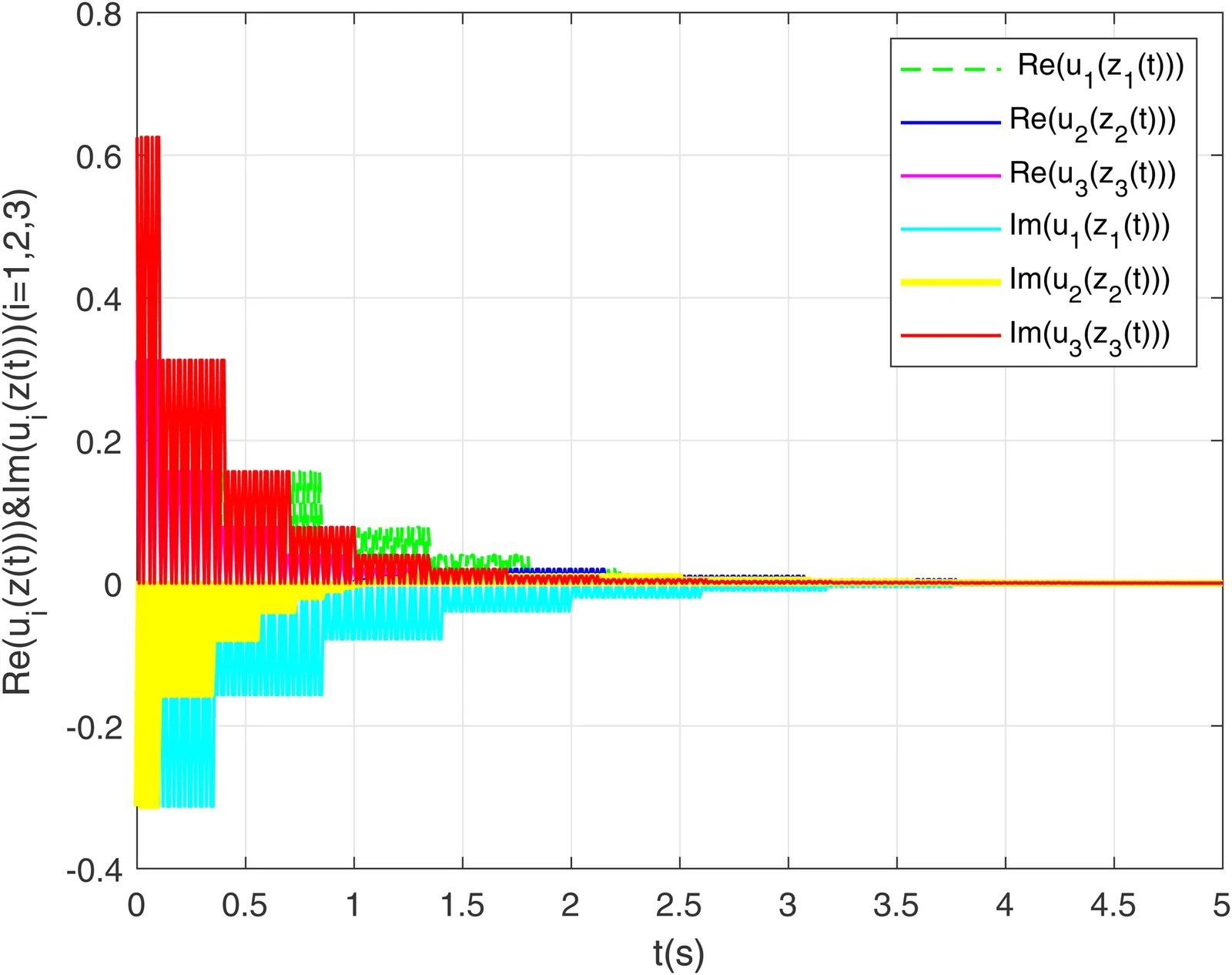
Inputs of real and imaginary parts for the controller (2).

**Fig 4:**
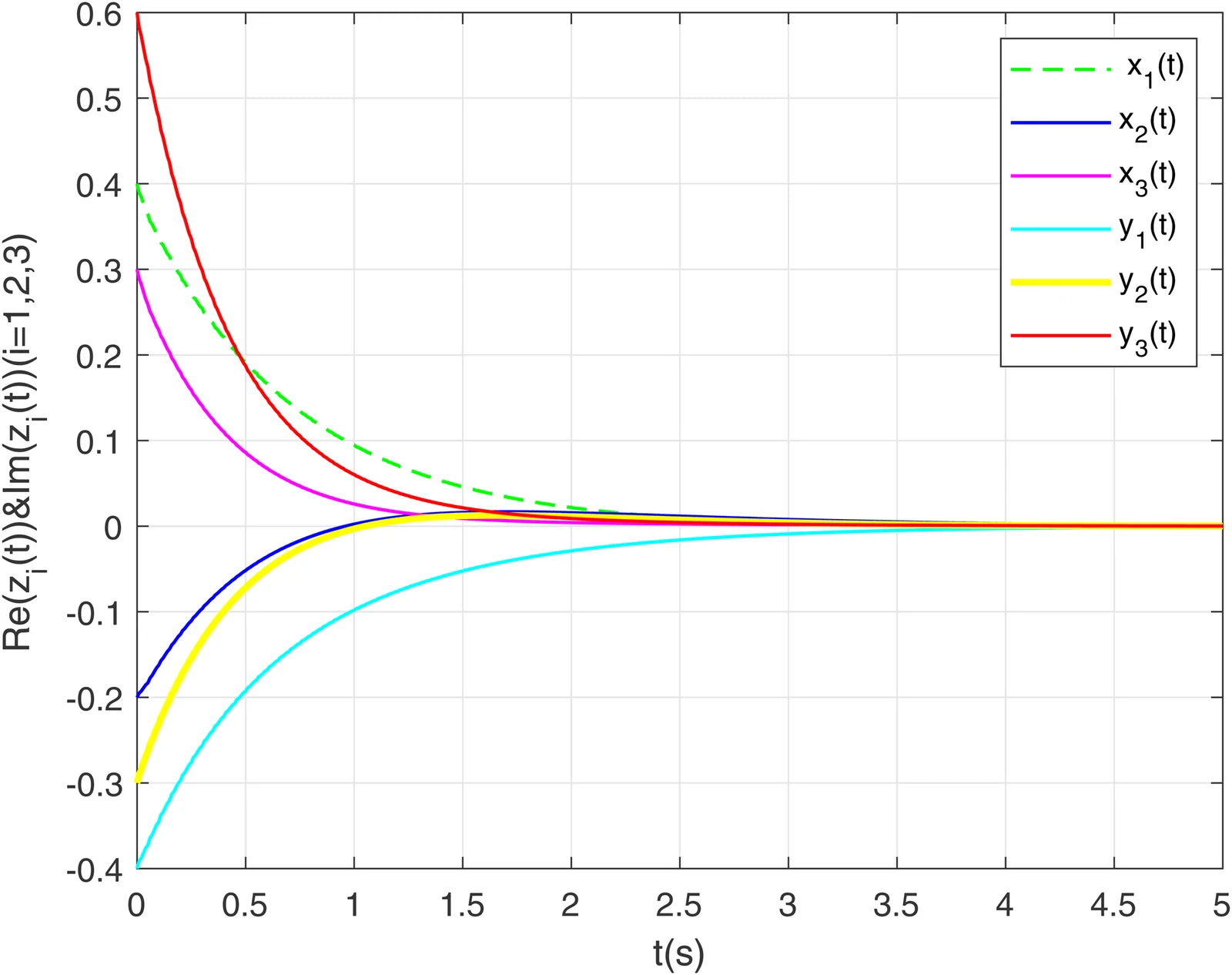
State trajectories of real and imaginary parts for system (6).

Through Theorem 1, system (1) is exponential stabilized with controller (2). Fig 3–Fig 4 depict inputs of the controller *Re*(*u*(*z*(*t*)), *Im*(*u*(*z*(*t*))) and system state trajectories *Re*(*z*(*t*)), *Im*(*z*(*t*)), respectively. It indicates that the network states approach to zero ultimately. Next, choose $h_{k}\in [0.01,0.05]$, $\Delta h_{k}=0.01$ and α=0.4. According to the designed one dimensional search algorithm, Table 2 is given which indicates the quantitative relationship between $h_{k}$ and $\overset{―}{\Psi }$. From Table 2, one can see that $\overset{―}{\sigma }$ and $\overset{―}{\Psi }$ become smaller as the transmission length $h_{k}$ becomes larger.

In Theorem 2, select $h_{1}=h_{2}=0.08,\;\alpha =0.4,\;\sigma =0.4,\;\rho =0.5,\;\beta =0.5,\;\Psi =0.4$, then the feedback control gain can be obtained as:


$$\begin{array}{c} \varrho =2[\sigma (1-\Psi )-\alpha \Psi ]=0.16>0, \end{array}$$

$$\begin{array}{c} K=\left[\begin{array}{ccc} -0.4313 & -0.0480 & 0.0671\\ -0.0285 & -0.4613 & 0.0462\\ -0.0414 & -0.0037 & -0.5329 \end{array}\right]. \end{array}$$ (27)

**Fig 5:**
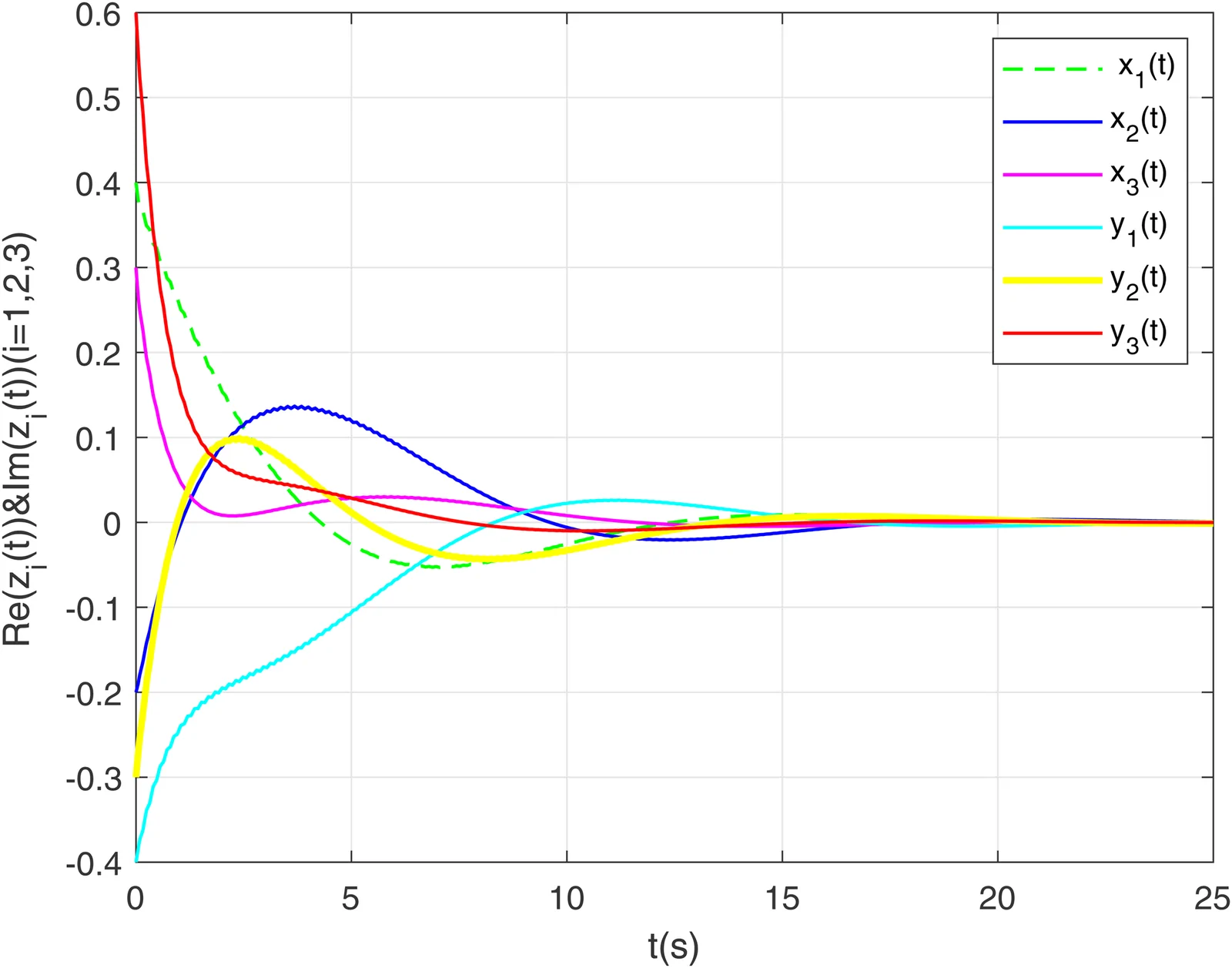
State trajectories of system (6) with QIST controller.

**Fig 6:**
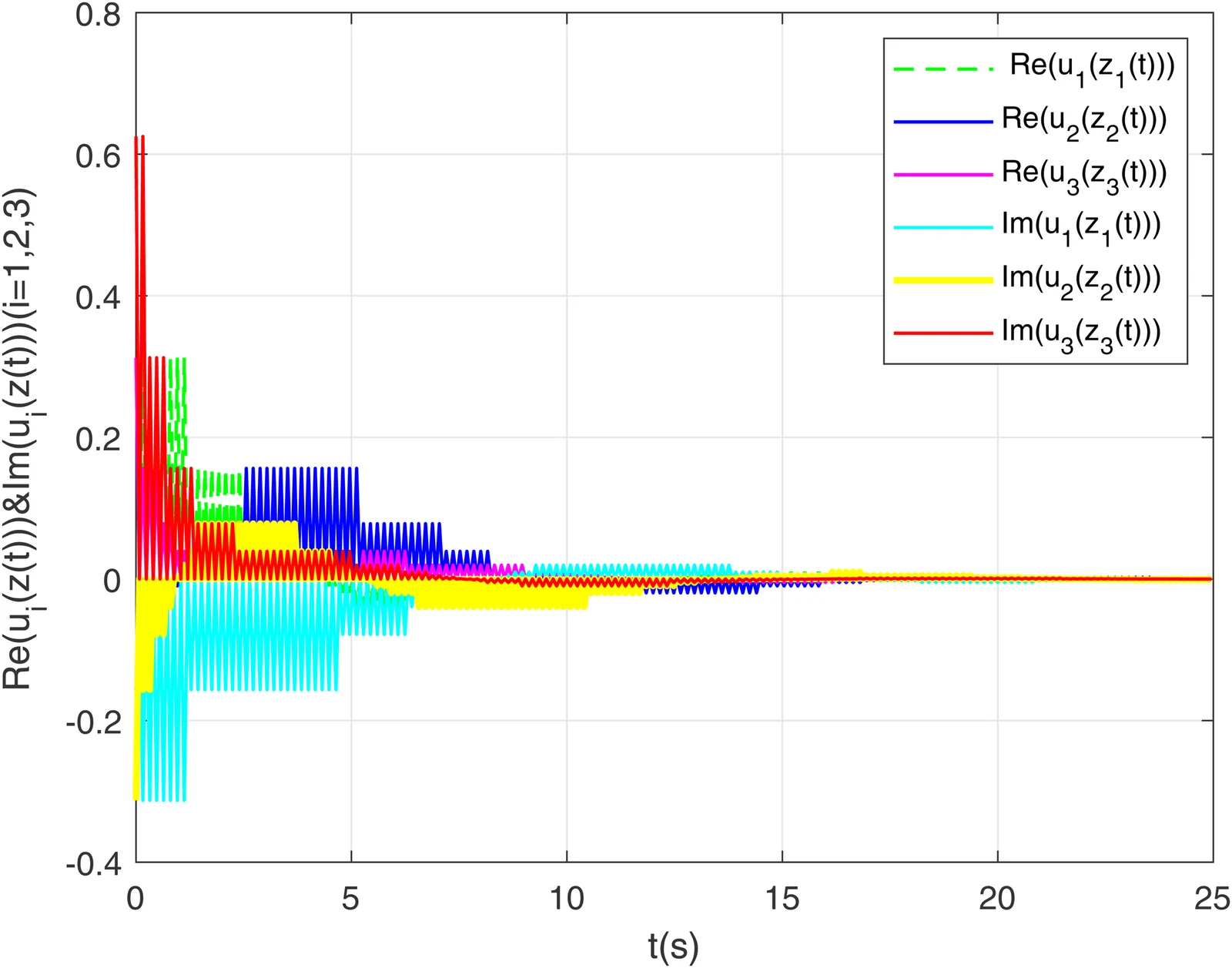
Inputs of real and imaginary parts for QIST controller.

Then, system (1) is exponential stabilized and the time evolutions of real and imaginary parts for the state *z*(*t*) and the inputs for the controller *u*(*z*(*t*)) of controlled system (6) are depicted in Fig 5 and Fig 6. One selects ρ=1, and the quantizer densities δ=0 which means there is no quantization. The QIST scheme is reduced to IST scheme. Then by the Theorem 2, the corresponding controller gains are


$$\begin{array}{c} \hat{K}=\left[\begin{array}{ccc} -0.2417 & 0.0031 & 0.0001\\ 0.0062 & -0.2425 & 0.0019\\ -0.0178 & -0.0336 & -0.2385 \end{array}\right]. \end{array}$$ (28)

**Fig 7:**
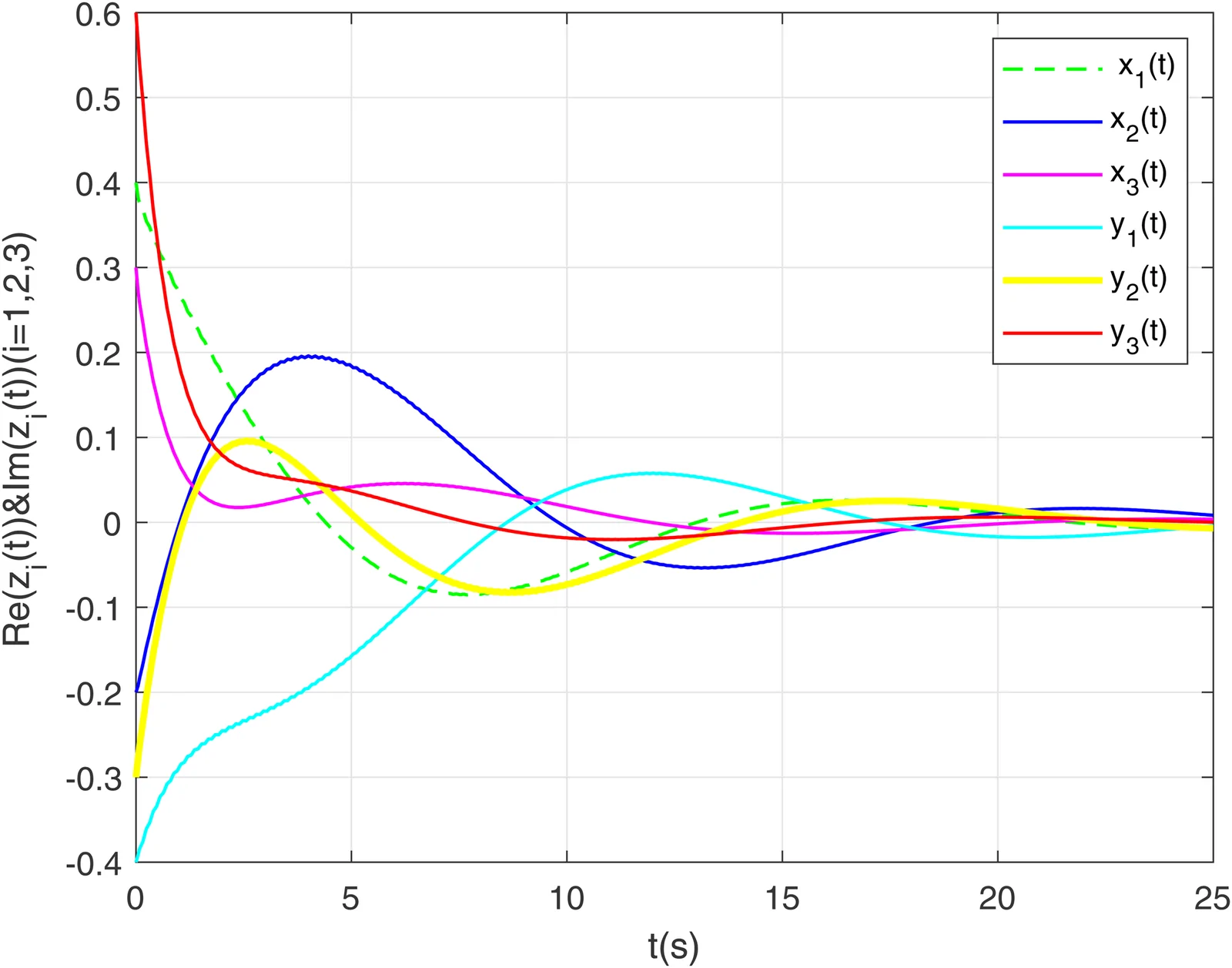
State trajectories of system (6) with IST controller.

**Fig 8:**
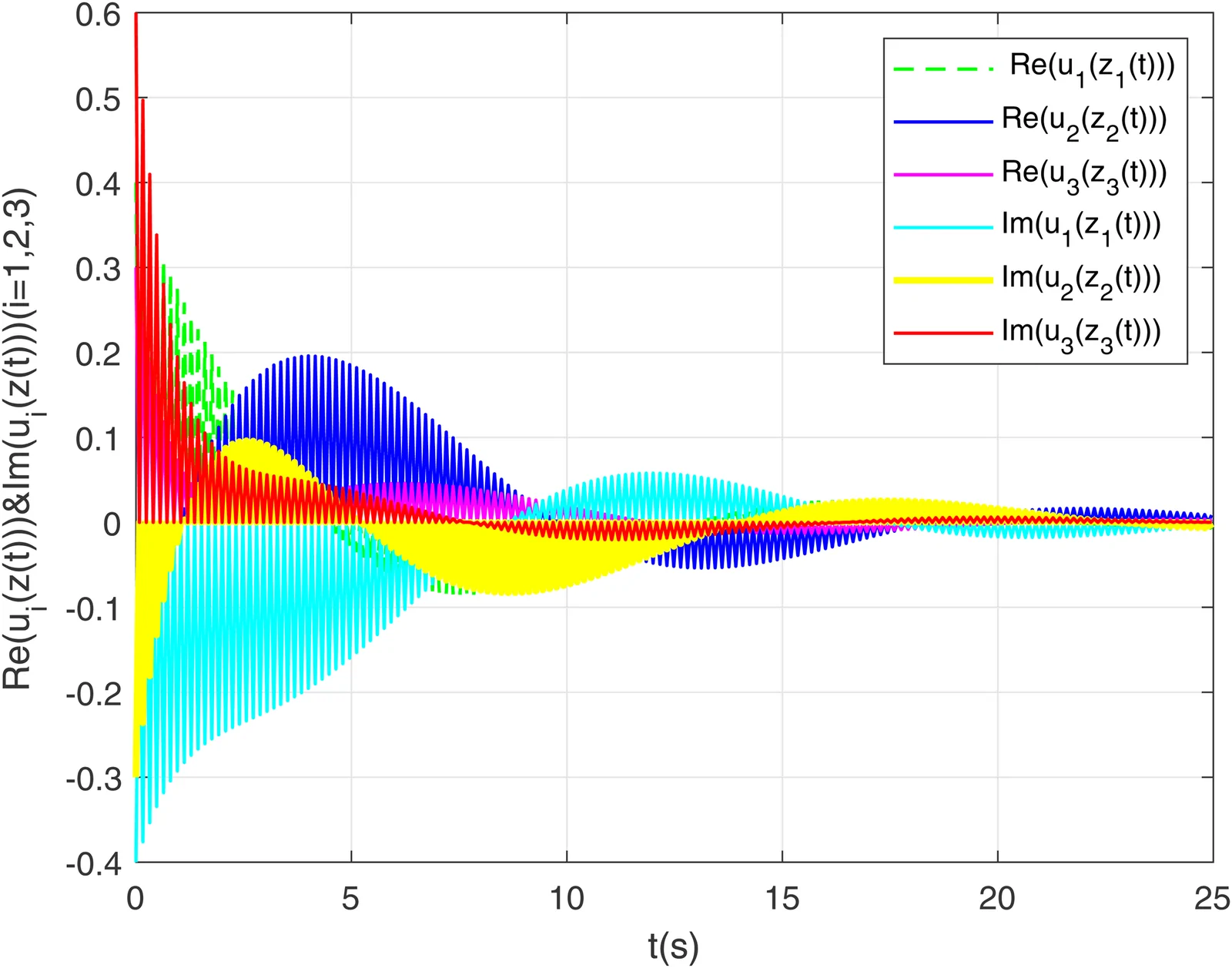
Inputs of real and imaginary parts for IST controller.

The state trajectories of the system (1) with the control gains (28) are given in Fig 7 and Fig 8. Comparing the state trajectories under QIST and IST in Fig 5 and Fig 7, both schemes achieve stabilization with the states converging to zero. However, the proposed QIST scheme exhibits faster decay, smaller oscillation amplitude, and a smoother dynamic response. In contrast, the IST scheme shows larger overshoot and longer-term oscillations, leading to a slower convergence rate. Meanwhile, the introduction of quantization in QIST further reduces the amount of transmitted data. Thus, QIST achieves superior or comparable control performance while significantly reducing communication bandwidth usage and actuator workload, demonstrating its dual advantages in control performance and resource efficiency.

Comparing the control input trajectories of QIST controller and IST controller in Fig 6 and Fig 8, both schemes achieve the control objective of converging the input to zero. However, the control input under QIST presents a stepwise discrete update form, which is set to zero during the intermittent rest intervals, significantly reducing the actuator operating time. Meanwhile, the quantization mechanism greatly reduces the amount of data transmitted each time, leading to lower communication bandwidth usage. In contrast, the control input under IST fluctuates continuously throughout the simulation, with almost no rest time for the actuator, and the transmission of unquantized continuous values incurs higher communication overhead. This fully demonstrates the advantage of QIST in reducing resource consumption while maintaining satisfactory control performance.

Now, one defines two indexes related to control cost in practice:(1) the total length of the actuator rest interval *L*;(2) the minimum switching times $S_{\min}$ of electronic devices. Take the simulation time *t* = 25*s*. With the obtained feedback gain *K*, one ob*t*ains that $L=25\times \overset{―}{\Psi }=12.5s$ and $S_{\min}=25/h_{2}\approx 312$. With fixed $\overset{―}{\Psi }$, one can analyse the quantitative relationship between *L* and $S_{\min}$ via the one-dimensional search algorithm to find $h_{k}$. Take $\overset{―}{\Psi }\in [30\%,70\%]$ and $\Delta \overset{―}{\Psi }=10\%$. Then, Table 3 is obtained and it reveals that the larger $\overset{―}{\Psi }$ and $h_{k}$ indicate the actuator has more rest time and electronic equipments has less switching times, respectively. Thus, there is a balance between the switch losses of electronic equipments and the control cost.

## 5. Conclusion

A QIST scheme is designed in this paper to address the exponential stability issue of DCNNs. A QIST protocol is proposed to reduce the data transmission load. A transmission-interval-dependent and switched Lyapunov functional is designed to derive less conservative exponential stability conditions. Furthermore, it reveals the quantitative relationship between the duty cycle of the rest intervals and the transmission length. It carries out one representative example with numerical simulation to demonstrate the efficiency of the suggested QIST protocol.

## Supporting information

S1 Dataset This file is used to generate Fig 2. (XLSX)

S2 Dataset This file is used to generate Fig 3. (XLSX)

S3 Dataset This file is used to generate Fig 4. (XLSX)

S4 Dataset This file is used to generate Fig 5. (XLSX)

S5 Dataset This file is used to generate Fig 6. (XLSX)

S6 Dataset This file is used to generate Fig 7. (XLSX)

S7 Dataset This file is used to generate Fig 8. (XLSX)
