# Supplementary material for: Sampled-data exponential stabilization for delayed complex valued neural networks with quantized intermittent communication
Source: PLoS One. 2026 Sep 9;21(9):e0357795. doi: 10.1371/journal.pone.0357795 (PMC13557402; doi:10.1371/journal.pone.0357795)
Supplement: Supplementary file 1 [file pone.0357795.s009.pdf]

Authors' Responses (Ms. Ref. No.: PONE-D-26-02182)

Ms. Ref. No.: PONE-D-26-02182
Title: Sampled-data exponential stabilization for delayed complex valued neural networks with quantized intermittent communication
Authors: Jie Li, Xiaohong Wang*

Reply to the Academic Editor

The authors would like to thank the academic editor, and the reviewers very much for their valuable comments and suggestions, which lead to significant improvement of the quality and presentation of the manuscript. We have seriously considered these comments and suggestions, and carefully revised the manuscript accordingly. In what follows, we will provide a detailed point-to-point response with regard to the comments. The revisions are highlighted in blue fonts in the revised paper.

0)**Your comment and suggestion:** *This paper is relevant to the topic and possesses certain innovative points, but in order to meet the publication standards, it still requires certain modifications The reviewer's comments primarily focused on the following aspects:*
1. The simulation is not perfect enough.
2. Lack of parameter analysis content such as data sensitivity analysis.
3. Certain modifications are needed in terms of format and language.
Welcome to continue submitting after revision.

Our reply: Thanks the academic editor for the time and efforts in handling the paper and for providing the opportunity for us to revise the manuscript. In light of the comments of reviews, we have made a major and very careful revision of this paper. The detailed point-by-point responses are as follows.

Comment 1: The simulation is not perfect enough.

Response: Thank you very much for pointing out the deficiencies in the simulation part. We fully recognize that the original simulation lacked sufficient depth and comparative analysis. Accordingly, we have substantially supplemented and optimized the numerical simulation section (Section 4) in the revised manuscript. The specific improvements are as follows:

1. **Added comparative study:** We have added a detailed comparison with the scheme without quantization (i.e., the intermittent sampled-data transmission (IST) scheme). In the new Figures 4 and 5, we respectively show the comparisons of system state trajectories and control inputs under the proposed QIST scheme and the IST scheme. The results clearly demonstrate that the proposed QIST scheme exhibits significant advantages in convergence speed, oscillation amplitude, and communication resource consumption.
2. **Explicitly provided key parameter values:** At the beginning of the simulation, we now explicitly provide all system and control parameter values, for example: activation functions $f(z(t)) =$

$g(z(t)) = \tanh(z(t))$, time-varying delay $\tau(t) = \frac{t}{10(1+e^t)}$ (with upper bound $\tau_M = 0.1$ and derivative bound $\dot{\tau}(t) \leq 0.025$), quantization density $\rho = 0.5$ (corresponding to quantization error bound $\delta = \frac{1}{3}$), and control gain $K = \text{diag}\{-2, -2, -2\}$. Thus, the simulation setup is fully reproducible.

3. **Enhanced analysis depth:** We not only verify stability but also further analyze the practical benefits of the QIST protocol in engineering applications. Two indices related to control cost are defined: the total length of the actuator rest interval L and the minimum switching times $S_{\min}$ of electronic devices. Quantitative results are provided (e.g., for simulation time $t = 25\,s$ and $\overline{\Psi} = 50\%$, we have $L = 12.5\,s$ and $S_{\min} = 312$), thereby more comprehensively demonstrating the resource-saving advantages of the proposed control scheme.

These modifications make the simulation results more convincing and provide stronger support for the conclusions of the paper.

Comment 2: Lack of parameter analysis content such as data sensitivity analysis.

Response: Thank you very much for this important suggestion. We fully agree that an in-depth parameter sensitivity analysis is crucial for evaluating the robustness and practicality of the control scheme. In the revised manuscript, we have systematically added such content:

1. **Revealed quantitative relationships among key parameters:** In Section 3 and the numerical simulation section (Tables 1 and 2), using the proposed one-dimensional search algorithm, we analyze in detail the quantitative relationships between the transmission length h_k, the duty cycle Ψ, the decay rates σ and α.
   - **Table 1** clearly shows: as the transmission length h_k increases from 0.01 to 0.05, the maximum allowable duty cycle $\overline{\Psi}$ decreases monotonically from 57.9% to 50%. This finding is one of the important theoretical contributions of our paper, revealing the design trade-off that a larger transmission interval implies a smaller proportion of rest time that the system can tolerate.
   - **Table 2** analyzes, for different desired duty cycles $\overline{\Psi}$, the required transmission length h_k and the corresponding minimum switching times $S_{\min}$. For example, when the actuator is expected to rest for 70% of the time, the transmission interval needs to be set to $h_2 = 0.08$, resulting in 312 switching times. This provides clear guidance for engineers to select parameters according to actual hardware switching losses and energy-saving requirements.
2. **Added discussion on parameter coupling (Remark 5):** We have added a new Remark 5 to specifically discuss the coupling relationships among the parameters h_k, Ψ, σ, and α. It is noted that although a closed-form analytical expression is unavailable, a systematic numerical search algorithm can determine the feasible parameter boundaries, and Tables 1 and 2 are the concrete results obtained by this method.
3. **Quantified the effect of quantization density:** In the simulation, we take $\rho = 0.5$ ($\delta = 1/3$) and compare it with the case $\rho = 1$ ($\delta = 0$, i.e., no quantization) in the IST scheme, indirectly showing the impact of different quantization densities on control performance.

With these additions, the parameter sensitivity analysis is now much more comprehensive, and the connection between theoretical results and engineering practice is significantly strengthened.

Comment 3: Certain modifications are needed in terms of format and language.

Response: Thank you very much for your careful review regarding format and language. We have thoroughly polished and revised the entire manuscript according to this comment:

1. **Language:** We have checked the English expression sentence by sentence, correcting grammatical errors, unnatural phrasing, and unclear logical connections. For example:
   - The redundant or unclear sentences in the abstract and introduction have been reorganized and refined.
   - Some inconsistencies in symbols and expressions in the proof section have been corrected.
   - The use of tenses and voices throughout the manuscript has been unified.
2. **Format:**
   - We have checked and unified the fonts and formats of all mathematical symbols (e.g., vectors, matrices, sets).
   - All abbreviations are provided with their full definitions at their first occurrence.
   - The citations of all figures, tables, equations, and references have been checked to ensure they are correct.
   - The page layout and heading hierarchy have been normalized according to the journal's final requirements.

We believe that after these modifications, the fluency and formatting of the manuscript have met the publication standards of the journal.

Once again, we sincerely thank the editors and reviewers for their hard work and valuable suggestions. We hope that the revised manuscript meets your expectations and look forward to your further review.

Reply to Reviewer 1

The authors wish to express their appreciation to the reviewer for insightful comments and useful suggestions. Without the reviewer's help, the paper cannot reach the present much improved level.

0)**Your comment and suggestion:** *In this paper, a quantized intermittent sampled-data transmission scheme is proposed for delayed complex-valued neural networks. The study provides stability criteria based on a transmission-interval-dependent Lyapunov functional. However, there are still some issues that require further clarification and consideration by the authors. Therefore, my recommendation is Major Revision.*

Our reply: Thanks the reviewer very much for the encouraging comments and the positive recommendation. We hope the revised paper is satisfactory for publication.

Question 1: *The paper only illustrates the system state trajectories under the proposed control method. To demonstrate the advantages of QIST, it is recommended to include comparative simulations.*

Our reply: Thank you for this important suggestion. We agree that comparative simulations are essential to clearly demonstrate the advantages of the proposed Quantized Intermittent Sampled-data Transmission (QIST) protocol over existing methods. In the revised manuscript, we have added a set of comparative simulation results in Section 4 (Numerical Simulation). Specifically, we compare the proposed QIST-based control scheme with the conventional Intermittent Sampled-data Transmission (IST) scheme without quantization (i.e., $\delta = 0$). The key comparisons include:

- **State trajectories (Fig 5, Fig 7):** Both QIST and IST achieve exponential stabilization, but QIST exhibits faster convergence, smaller oscillation amplitude, and smoother transient response.
- **Control input trajectories (Fig 6, Fig 8):** Under QIST, the control input is updated in a stepwise, discrete manner and remains zero during rest intervals, significantly reducing actuator operation time. In contrast, IST requires continuous control input updates with almost no rest,

leading to higher energy consumption.

- **Resource efficiency:** With quantization, QIST reduces the amount of transmitted data per sampling instant, thereby lowering communication bandwidth usage. A quantitative analysis of the total actuator rest time L and minimum switching times $S_{\min}$ is also provided in Table 2.

These comparative results clearly demonstrate that the proposed QIST protocol not only achieves satisfactory control performance but also offers significant advantages in terms of communication load reduction, actuator rest time, and switching frequency of electronic devices. We believe these additions effectively address your concern.

We have marked all modifications in the revised manuscript for your convenience. We greatly appreciate your insightful comment, which has helped us substantially strengthen the technical contribution of this work.

Question 2: *The experimental results depend on the selection of parameters. Currently, the tables only provide numerical observations from specific trials, which lacks theoretical analysis of parameter sensitivity. It is recommended to derive mathematical formulas to describe the boundary relationships between parameters.*

Our reply: Thanks for your valuable comments. In this paper, the parameters involved such as the transmission length h_k, the duty cycle of rest intervals Ψ, and the decay rates σ and α are interrelated through a set of feasibility conditions expressed as linear matrix inequalities (LMIs). These LMIs do not admit an explicit closed-form analytical solution for the parameter boundaries. Instead, the feasible parameter regions are implicitly defined by the solvability conditions of these LMIs, which inherently depend on the system matrices and the chosen Lyapunov functional.

Given the complexity and nonlinear coupling among parameters, the relationships between them are essentially *quantitative* rather than *analytical*, and thus their boundaries can only be determined through numerical simulation and iterative feasibility checks, such as the one-dimensional search algorithm provided in Section 3 (see Step 1–Step 4). The numerical results presented in Tables 1 and 2 are not merely "specific trials," but rather representative points obtained by systematically scanning parameter ranges at a prescribed resolution (e.g., $\Delta h_k = 0.01$, $\Delta\overline{\Psi} = 10\%$), which delineate the maximal admissible $\overline{\Psi}$ for a given h_k and vice versa. These tables therefore serve as a practical mapping of the parameter boundary-a form of "quantitative relational characterization."

To further strengthen the theoretical insight, we note that inequality (11) provides a necessary algebraic relation:

$$\rho = 2[\sigma(1-\Psi) - \alpha\Psi] > 0,$$

which establishes a linear boundary between σ, α, and Ψ. However, the exact feasible region for (h_k, Ψ) must still be determined by LMI feasibility, as additional constraints (7)–(10) impose nonlinear coupling with h_k and the system dynamics. Hence, we maintain that a fully analytical formula for the boundary is not attainable, but the proposed numerical search combined with LMI solvers provides a rigorous and practical determination of the parameter margin.

In response to the reviewer's suggestion, we have added a remark in the revised manuscript (see Remark 5) clarifying the quantitative nature of the parameter relationships and explicitly stating that the feasible boundaries are obtained via the described numerical search procedure, which is standard in LMI-based control design for sampled-data and intermittent control systems. We believe this addresses the concern while being consistent with the methodological limitations of the approach.

Question 3: *The matrices in the theorem are of high dimensionality. The authors should provide a discussion regarding the issue of computational complexity.*
Our reply: We thank the reviewer for this insightful comment. We have added the Remark 4 on computational complexity in the revised manuscript to address this concern.

Question 4: *Please standardize the terminology by replacing all instances of "thesis" with "paper"*
Our reply: We apologize for this inconsistency. In the revised manuscript, we have replaced all instances of thesis with paper throughout the paper.

Thank you for your constructive comments and your great help which can improve the clarity of this paper, and we hope the revised paper is satisfactory for publication.

Reply to Reviewer 2

The authors wish to express their appreciation to the reviewer for insightful comments and useful suggestions. Without the reviewer's help, the paper cannot reach the present much improved level.

Question 1: *The authors can rewrite the abstract by adding the achieved results.*
Our reply: Many thanks for the reviewer for this constructive suggestion. In the revised manuscript, we have rewritten the abstract to more explicitly highlight the main results achieved in this paper.

Question 2: *A few of the equations don't have equation number.*
Our reply: We thank the reviewer for this careful observation. In our manuscript, only the key equations that are directly referenced in the main results or used for subsequent derivations (such as the system model, controller protocol, main matrix inequalities in the theorems, and final stability conditions) have been assigned equation numbers. The unnumbered equations, including certain intermediate algebraic manipulations, auxiliary inequalities in the derivation process, or transitional expressions, serve as supporting steps leading to the numbered key equations. This practice follows the common convention in academic writing to avoid excessive numbering that might distract readers from the core results, while keeping the presentation concise. If the reviewer deems necessary, we are happy to add numbers to some of the important intermediate equations or provide clarification in the revised manuscript. Once again, we appreciate the reviewer's valuable suggestion.

Question 3: *It is very difficult to understand Theorem 1! The authors can modify it so that readers can understand it easily.*
Our reply: Thanks for your valuable comments. We thank the reviewer for this constructive comment. We agree that Theorem 1, as originally presented, is dense and may be difficult for readers to follow due to the high dimensionality of the matrices and the large number of entries. To improve readability, we have revised Theorem 1 in the updated manuscript.

Question 4: *The authors can add more information about simulation setting.*
Our reply: Many thanks for your interesting question. In the revised manuscript, we have expanded the description of the simulation setting in Section 4. Specifically, we now provide:

- The exact numerical values of all system matrices D, A, B and the nonlinear functions $f(\cdot)$, $g(\cdot)$.
- The initial values $\phi(s)$ and the time-varying delay $\tau(t) = \frac{e^t}{10(1+e^t)}$, with $\tau_M = 0.1$ and $\dot{\tau}(t) \leq 0.025 < 1$.
- The quantizer density: $\rho = 0.5$, initial quantization $u_0 = 0.2$, and the quantization parameter

$\delta = \frac{1-\rho}{1+\rho} = \frac{1}{3}$.

Question 5: *More simulation results can be added.*

Our reply: Thanks for the valuable comment. We have supplemented more simulation results in the revised manuscript. We add comparative simulations between the proposed QIST scheme and the conventional IST scheme. The new simulation results further demonstrate that our method has better dynamic performance and lower communication resource consumption, and the relevant figures and analysis have been added in Section 4.

Question 6: *More recent paper can be added to improve the literature.*

Our reply: Thanks very much for the reviewers carefully reading and suggestions. According to your comments, we have added some recent papers in revised manuscript.

Thank you for your constructive comments and your great help which can improve the clarity of this paper, and we hope the revised paper is satisfactory for publication.
