# Supplementary material for: Sampled-data exponential stabilization for delayed complex valued neural networks with quantized intermittent communication
Source: PLoS One. 2026 Sep 9;21(9):e0357795. doi: 10.1371/journal.pone.0357795 (PMC13557402; doi:10.1371/journal.pone.0357795)
Supplement: Supplementary file 2 [file pone.0357795.s010.pdf]

Authors' Responses (Ms. ID: PONE-D-26-02182R1)

Ms. ID: PONE-D-26-02182R1
Title: Sampled-data exponential stabilization for delayed complex valued neural networks with quantized intermittent communication
Authors: Jie Li, Xiaohong Wang*

The authors would like to thank the Editor-in-Chief, the Associate Editor-in-Chief, the Associate Editor and the reviewers very much for their valuable comments and suggestions, which lead to significant improvement of the quality and presentation of the manuscript. We have seriously considered these comments and suggestions, and carefully revised the manuscript accordingly. In what follows, we will provide a detailed point-to-point response with regard to the comments.

Reply to Reviewer 2

The authors wish to express their appreciation to the reviewer for insightful comments and useful suggestions. Without the reviewer's help, this paper cannot reach the present much improved level.

0)**Your comment and suggestion:** *I would like to give thanks to the authors for addressing the comments that were given in the previous round.*

Our reply: We sincerely thank the reviewer for the positive feedback and for taking the time to evaluate our revised manuscript. We are grateful for the recognition of our revisions.

Reply to Reviewer 3

The authors wish to express their appreciation to the reviewer for insightful comments and useful suggestions. Without the reviewer's help, this paper cannot reach the present much improved level. According to your comments and suggestions, we have made a very careful revision. The revisions are highlighted in blue fonts in the revised version.

Your comment and suggestion: *This paper presents a quantized intermittent sampled-data transmission protocol to solve the sampled-data exponential stabilization problem for delayed complex-valued neural networks. The paper is original and technically interesting. However, it can be revised to further improve its readability. Some discussions are not clearly stated which may hinder the readers' understanding.*

Our reply: Thanks the reviewer for having brought this valuable comments to our paper. We have carefully addressed each point as detailed below.

Question 1: *The description of the paper's contributions in the introduction is vague.*

Our reply: We thank the reviewer for this valuable comment. We have revised the introduction to present the contributions of this paper more clearly and explicitly. Specifically, we have restructured the

contribution statement to highlight three distinct aspects:

(a) Different from existing quantized intermittent sampled-data transmission (QIST) schemes that are only developed for real-valued neural networks, this work establishes a complete QIST control framework for delayed complex-valued neural networks (DCNNs). A complex-domain state feedback controller is designed, which significantly cuts network transmission frequency and actuator running time to improve energy efficiency under limited communication bandwidth.

(b) A novel transmission-interval-dependent switched Lyapunov functional is specially constructed to match the two-mode switching characteristic of the QIST protocol (transmission interval $[t_k, s_k)$ and rest interval $[s_k, t_{k+1})$). Compared with conventional Lyapunov-Krasovskii functionals for intermittent control, the proposed functional maintains continuity at switching instants and imposes much milder constraints on Lyapunov matrices, which fully exploits sampled state information to reduce conservatism.

(c) Combining sector-bounded quantization characterization and advanced integral inequality techniques, two less-conservative exponential stabilization criteria are derived in the form of complex-valued LMIs. For the first time, our conditions quantitatively reveal the explicit coupling relationship among exponential convergence rate, quantization density, maximum transmission length and rest-interval duty cycle. A one-dimensional search algorithm is further provided to solve the admissible upper bound of the duty cycle.

Question 2: *The extensibility of the proposed method is not discussed. For instance, can this method apply to large-scale systems, as considered in "decentralized adaptive delayed control of large-scale nonlinear systems with random couplings and biased measurements"?*

Our reply: We thank the reviewer for pointing out this important missing discussion on method extensibility and scalability to large-scale systems. We have incorporated this discussion as a newly added Remark 5 at the end of Section 3 (Stability Analysis) in the revised manuscript.

Question 3: *Why is the quantization scheme in (3) chosen? Can other types of quantizers also be applied?*

Our reply: We thank the reviewer for this question. We have clarified the motivation for choosing the logarithmic quantizer in (3) by adding a new explanatory paragraph immediately after equation (3) in Section 2.

The logarithmic quantizer in (3) is adopted for three main reasons. First, it admits a convenient sector-bound description $q(v) - v = \Delta(t)v$ with $||\Delta(t)|| \leq \delta$, which enables the quantization error to be incorporated into the subsequent LMI-based stability analysis in a tractable manner. Second, the quantization density parameter $\rho \in (0, 1)$ provides a direct trade-off between quantization accuracy and communication bit rate, which is crucial for capacity-limited channels. Third, this type of quantizer has been widely validated in [45]–[50] and is standard for neural network systems with quantized feedback. It is worth noting that other quantizer types, such as uniform quantizers, dynamic quantizers with adjustable scaling, or hysteresis quantizers, could also be considered in principle. However, uniform quantizers generally lack the sector-bound property that facilitates LMI-based design, while dynamic and hysteresis quantizers would introduce additional dynamics or switching logic that complicate the stability analysis. Extending the proposed framework to accommodate these alternative quantization schemes is an interesting direction for future investigation.

Question 4: *The statement in Theorem 1 that the LMIs hold for arbitrary real matrices U, M, W, and N seems questionable.*

Our reply: We sincerely appreciate the reviewer for pointing out this critical wording error. The term

"arbitrary" was mistakenly used in the original manuscript, which leads to misleading interpretation. Matrices U, M, W, N are auxiliary free-weighting matrices (decision variables) introduced in the stability analysis via the free-weighting matrix technique. The correct mathematical meaning is that there exists a set of suitable matrices U, M, W, N such that the LMIs are satisfied, rather than the LMIs hold for all arbitrary choices of U, M, W, N. We have revised Theorem 1 in the revised manuscript accordingly, removing the incorrect word "arbitrary" and rephrasing the description of these auxiliary matrices to avoid confusion.

Question 5: *The resolution of Fig. 1 is low.*

Our reply: We have regenerated Fig. 1 from the original Visio source file. The figure is exported as lossless 600 dpi TIFF file complying with PLOS ONE standards, eliminating blurriness caused by low resolution. All other figures in the manuscript have also been rechecked and re-exported with high resolution.

Thank you again for your constructive comments and your great help which improve the clarity of this paper, and we hope the revised paper is satisfactory for publication.

Additional Revisions Addressing Journal Requirements

In addition to the above point-by-point responses, we have also addressed the following journal-specific requirements:

Data Availability Statement: We have revised the Data Availability Statement to ensure full compliance with PLOS ONE's data policy. All data underlying the findings are fully available within the manuscript and its supporting information.

Reference List Verification: We have carefully checked our reference list to ensure that it is complete, correct, and does not contain any retracted articles. All cited references are appropriate and up to date.

ORCID Verification: The corresponding author's ORCID iD has been verified in the submission system. We have also encouraged all coauthors to verify their ORCID iDs before final acceptance.

Language and Grammar: We have carefully proofread the entire manuscript to correct any typographical or grammatical errors.
