# Supplementary material for: Sampled-data exponential stabilization for delayed complex valued neural networks with quantized intermittent communication
Source: PLoS One. 2026 Sep 9;21(9):e0357795. doi: 10.1371/journal.pone.0357795 (PMC13557402; doi:10.1371/journal.pone.0357795)
Supplement: Peer review history

# Peer review: Decision Letter 0

**DOI:** 10.1371/journal.pone.0357795.r001

**Authors:** Neng Ye

3 Mar 2026

Dear Dr. wang,

Thank you for submitting your manuscript to PLOS ONE. After careful consideration, we feel that it has merit but does not fully meet PLOS ONE’s publication criteria as it currently stands. Therefore, we invite you to submit a revised version of the manuscript that addresses the points raised during the review process.

Please submit your revised manuscript by Apr 17 2026 11:59PM. If you will need more time than this to complete your revisions, please reply to this message or contact the journal office at plosone@plos.org. When you're ready to submit your revision, log on to https://www.editorialmanager.com/pone/ and select the 'Submissions Needing Revision' folder to locate your manuscript file.

- A letter that responds to each point raised by the academic editor and reviewer(s). You should upload this letter as a separate file labeled 'Response to Reviewers'.
- A marked-up copy of your manuscript that highlights changes made to the original version. You should upload this as a separate file labeled 'Revised Manuscript with Track Changes'.
- An unmarked version of your revised paper without tracked changes. You should upload this as a separate file labeled 'Manuscript'.

If you would like to make changes to your financial disclosure, please include your updated statement in your cover letter. Guidelines for resubmitting your figure files are available below the reviewer comments at the end of this letter.

If applicable, we recommend that you deposit your laboratory protocols in protocols.io to enhance the reproducibility of your results. Protocols.io assigns your protocol its own identifier (DOI) so that it can be cited independently in the future. For instructions see: https://journals.plos.org/plosone/s/submission-guidelines#loc-laboratory-protocols. Additionally, PLOS ONE offers an option for publishing peer-reviewed Lab Protocol articles, which describe protocols hosted on protocols.io. Read more information on sharing protocols at https://plos.org/protocols?utm_medium=editorial-email&utm_source=authorletters&utm_campaign=protocols.

We look forward to receiving your revised manuscript.

Kind regards,

Neng Ye

Academic Editor

PLOS One

Journal requirements:

When submitting your revision, we need you to address these additional requirements.

1. Please ensure that your manuscript meets PLOS ONE's style requirements, including those for file naming. The PLOS ONE style templates can be found at

https://journals.plos.org/plosone/s/file?id=wjVg/PLOSOne_formatting_sample_main_body.pdf and

https://journals.plos.org/plosone/s/file?id=ba62/PLOSOne_formatting_sample_title_authors_affiliations.pdf

2. Please note that PLOS One has specific guidelines on code sharing for submissions in which author-generated code underpins the findings in the manuscript. In these cases, we expect all author-generated code to be made available without restrictions upon publication of the work. Please review our guidelines at https://journals.plos.org/plosone/s/materials-and-software-sharing#loc-sharing-code and ensure that your code is shared in a way that follows best practice and facilitates reproducibility and reuse.

3. Please update your submission to use the PLOS LaTeX template. The template and more information on our requirements for LaTeX submissions can be found at http://journals.plos.org/plosone/s/latex.

4. We note that you have indicated that there are restrictions to data sharing for this study. PLOS only allows data to be available upon request if there are legal or ethical restrictions on sharing data publicly. For more information on unacceptable data access restrictions, please see http://journals.plos.org/plosone/s/data-availability#loc-unacceptable-data-access-restrictions.

Before we proceed with your manuscript, please address the following prompts:

a) If there are ethical or legal restrictions on sharing a de-identified data set, please explain them in detail (e.g., data contain potentially identifying or sensitive patient information, data are owned by a third-party organization, etc.) and who has imposed them (e.g., a Research Ethics Committee or Institutional Review Board, etc.). Please also provide contact information for a data access committee, ethics committee, or other institutional body to which data requests may be sent.

b) If there are no restrictions, please upload the minimal anonymized data set necessary to replicate your study findings to a stable, public repository and provide us with the relevant URLs, DOIs, or accession numbers. For a list of recommended repositories, please see

https://journals.plos.org/plosone/s/recommended-repositories. You also have the option of uploading the data as Supporting Information files, but we would recommend depositing data directly to a data repository if possible.

We will update your Data Availability statement on your behalf to reflect the information you provide.

If the reviewer comments include a recommendation to cite specific previously published works, please review and evaluate these publications to determine whether they are relevant and should be cited. There is no requirement to cite these works unless the editor has indicated otherwise.

Additional Editor Comments:

This paper is relevant to the topic and possesses certain innovative points, but in order to meet the publication standards, it still requires certain modifications

The reviewer's comments primarily focused on the following aspects:

1. The simulation is not perfect enough

2. Lack of parameter analysis content such as data sensitivity analysis

3. Certain modifications are needed in terms of format and language

Welcome to continue submitting after revision

[Note: HTML markup is below. Please do not edit.]

Reviewers' comments:

Reviewer's Responses to Questions

**Comments to the Author**

1. Is the manuscript technically sound, and do the data support the conclusions?

Reviewer #1: Partly

Reviewer #2: No

**********

2. Has the statistical analysis been performed appropriately and rigorously?

Reviewer #1: N/A

Reviewer #2: No

**********

3. Have the authors made all data underlying the findings in their manuscript fully available?

The PLOS Data policy

Reviewer #1: Yes

Reviewer #2: No

**********

4. Is the manuscript presented in an intelligible fashion and written in standard English?

Reviewer #1: No

Reviewer #2: No

**********

Reviewer #1: In this paper, a quantized intermittent sampled-data transmission scheme is proposed for delayed complex-valued neural networks. The study provides stability criteria based on a transmission-interval-dependent Lyapunov functional. However, there are still some issues that require further clarification and consideration by the authors. Therefore, my recommendation is Major Revision.

1. The paper only illustrates the system state trajectories under the proposed control method. To demonstrate the advantages of QIST, it is recommended to include comparative simulations.

2. The experimental results depend on the selection of parameters. Currently, the tables only provide numerical observations from specific trials, which lacks theoretical analysis of parameter sensitivity. It is recommended to derive mathematical formulas to describe the boundary relationships between parameters.

3. The matrices in the theorem are of high dimensionality. The authors should provide a discussion regarding the issue of computational complexity.

4. Please standardize the terminology by replacing all instances of "thesis" with "paper".

Reviewer #2: The comments are as follows:

(1) The authors can rewrite the abstract by adding the achieved results.

(2) A few of the equations don't have equation number.

(3) It is very difficult to ubderstand Theorem 1! The authors can modify it so that readers can understand it easily.

(4) The authors can add more information about simulation setting.

(5) More simulation results can be added.

(6) More recent paper can be added to improve the literature.

**********

what does this mean?). If published, this will include your full peer review and any attached files.

If you choose “no”, your identity will remain anonymous but your review may still be made public.

**Do you want your identity to be public for this peer review?**  For information about this choice, including consent withdrawal, please see our Privacy Policy

Reviewer #1: No

Reviewer #2: No

**********

[NOTE: If reviewer comments were submitted as an attachment file, they will be attached to this email and accessible via the submission site. Please log into your account, locate the manuscript record, and check for the action link "View Attachments". If this link does not appear, there are no attachment files.]

To ensure your figures meet our technical requirements, please review our figure guidelines: https://journals.plos.org/plosone/s/figures

You may also use PLOS’s free figure tool, NAAS, to help you prepare publication quality figures: https://journals.plos.org/plosone/s/figures#loc-tools-for-figure-preparation.

NAAS will assess whether your figures meet our technical requirements by comparing each figure against our figure specifications.

# Author comment: Author response to Decision Letter 1

**DOI:** 10.1371/journal.pone.0357795.r002

2 Jun 2026

The authors would like to thank the academic editor, and the reviewers very much for their valuable

comments and suggestions, which lead to significant improvement of the quality and presentation of the

manuscript. We have seriously considered these comments and suggestions, and carefully revised the

manuscript accordingly. In what follows, we will provide a detailed point-to-point response with regard to

the comments. The revisions are highlighted in blue fonts in the revised paper.

Attachment Submitted filename: Response to Reviewers.pdf

# Peer review: Decision Letter 1

**DOI:** 10.1371/journal.pone.0357795.r003

**Authors:** Gang Wang

9 Aug 2026

Dear Dr. wang,

Thank you for submitting your manuscript to PLOS ONE. After careful consideration, we feel that it has merit but does not fully meet PLOS ONE’s publication criteria as it currently stands. Therefore, we invite you to submit a revised version of the manuscript that addresses the points raised during the review process.

Please submit your revised manuscript by Sep 23 2026 11:59PM. If you will need more time than this to complete your revisions, please reply to this message or contact the journal office at plosone@plos.org. When you're ready to submit your revision, log on to https://www.editorialmanager.com/pone/ and select the 'Submissions Needing Revision' folder to locate your manuscript file.

- A letter that responds to each point raised by the academic editor and reviewer(s). You should upload this letter as a separate file labeled 'Response to Reviewers'.
- A marked-up copy of your manuscript that highlights changes made to the original version. You should upload this as a separate file labeled 'Revised Manuscript with Track Changes'.
- An unmarked version of your revised paper without tracked changes. You should upload this as a separate file labeled 'Manuscript'.

If you would like to make changes to your financial disclosure, please include your updated statement in your cover letter. Guidelines for resubmitting your figure files are available below the reviewer comments at the end of this letter.

If applicable, we recommend that you deposit your laboratory protocols in protocols.io to enhance the reproducibility of your results. Protocols.io assigns your protocol its own identifier (DOI) so that it can be cited independently in the future. For instructions see: https://journals.plos.org/plosone/s/submission-guidelines#loc-laboratory-protocols. Additionally, PLOS ONE offers an option for publishing peer-reviewed Lab Protocol articles, which describe protocols hosted on protocols.io. Read more information on sharing protocols at https://plos.org/protocols?utm_medium=editorial-email&utm_source=authorletters&utm_campaign=protocols.

As the corresponding author, your ORCID iD is verified in the submission system and will appear in the published article. PLOS supports the use of ORCID, and we encourage all coauthors to register for an ORCID iD and use it as well. Please encourage your coauthors to verify their ORCID iD within the submission system before final acceptance, as unverified ORCID iDs will not appear in the published article. *Only*  the individual author can complete the verification step; PLOS staff *cannot*  verify ORCID iDs on behalf of authors.

We look forward to receiving your revised manuscript.

Kind regards,

Gang Wang

Academic Editor

PLOS One

Journal Requirements:

If the reviewer comments include a recommendation to cite specific previously published works, please review and evaluate these publications to determine whether they are relevant and should be cited. There is no requirement to cite these works unless the editor has indicated otherwise.

Please review your reference list to ensure that it is complete and correct. If you have cited papers that have been retracted, please include the rationale for doing so in the manuscript text, or remove these references and replace them with relevant current references. Any changes to the reference list should be mentioned in the rebuttal letter that accompanies your revised manuscript. If you need to cite a retracted article, indicate the article’s retracted status in the References list and also include a citation and full reference for the retraction notice.

[Note: HTML markup is below. Please do not edit.]

Reviewers' comments:

Reviewer's Responses to Questions

**Comments to the Author**

Reviewer #2: All comments have been addressed

Reviewer #3: All comments have been addressed

**********

2. Is the manuscript technically sound, and do the data support the conclusions?

Reviewer #2: Partly

Reviewer #3: Yes

**********

3. Has the statistical analysis been performed appropriately and rigorously?

Reviewer #2: Yes

Reviewer #3: Yes

**********

4. Have the authors made all data underlying the findings in their manuscript fully available?

The PLOS Data policy

Reviewer #2: No

Reviewer #3: Yes

**********

5. Is the manuscript presented in an intelligible fashion and written in standard English?

Reviewer #2: Yes

Reviewer #3: Yes

**********

Reviewer #2: I would like to give thanks to the authors for addressing the comments that were given in the previous round.

Reviewer #3: This paper presents a quantized intermittent sampled-data transmission protocol to solve the sampled-data exponential stabilization problem for delayed complex-valued neural networks. The paper is original and technically interesting. However, it can be revised to further improve its readability. Some discussions are not clearly stated which may hinder the readers’ understanding. Some comments are listed as follows:

1. The description of the paper’s contributions in the introduction is vague.

2. The extensibility of the proposed method is not discussed. For instance, can this method apply to large-scale systems, as considered in “decentralized adaptive delayed control of large-scale nonlinear systems with random couplings and biased measurements”?

3. Why is the quantization scheme in (3) chosen? Can other types of quantizers also be applied?

4. The statement in Theorem 1 that the LMIs hold for arbitrary real matrices U, M, W, and N seems questionable.

5. The resolution of Fig. 1 is low.

**********

what does this mean?). If published, this will include your full peer review and any attached files.

If you choose “no”, your identity will remain anonymous but your review may still be made public.

**Do you want your identity to be public for this peer review?**  For information about this choice, including consent withdrawal, please see our Privacy Policy

Reviewer #2: No

Reviewer #3: No

**********

[NOTE: If reviewer comments were submitted as an attachment file, they will be attached to this email and accessible via the submission site. Please log into your account, locate the manuscript record, and check for the action link "View Attachments". If this link does not appear, there are no attachment files.]

To ensure your figures meet our technical requirements, please review our figure guidelines: https://journals.plos.org/plosone/s/figures

You may also use PLOS’s free figure tool, NAAS, to help you prepare publication quality figures: https://journals.plos.org/plosone/s/figures#loc-tools-for-figure-preparation.

NAAS will assess whether your figures meet our technical requirements by comparing each figure against our figure specifications.

# Author comment: Author response to Decision Letter 2

**DOI:** 10.1371/journal.pone.0357795.r004

20 Aug 2026

The authors would like to thank the Editor-in-Chief, the Associate Editor-in-Chief, the Associate Ed

itor and the reviewers very much for their valuable comments and suggestions, which lead to significant

improvement of the quality and presentation of the manuscript. We have seriously considered these com

ments and suggestions, and carefully revised the manuscript accordingly. In what follows, we will provide

a detailed point-to-point response with regard to the comments.

Reply to Reviewer 2

The authors wish to express their appreciation to the reviewer for insightful comments and useful sug

gestions. Without the reviewer’s help, this paper cannot reach the present much improved level.

0)Your comment and suggestion: I would like to give thanks to the authors for addressing the com

ments that were given in the previous round.

Our reply: We sincerely thank the reviewer for the positive feedback and for taking the time to evaluate

our revised manuscript. We are grateful for the recognition of our revisions.

Reply to Reviewer 3

The authors wish to express their appreciation to the reviewer for insightful comments and useful sug

gestions. Without the reviewer’s help, this paper cannot reach the present much improved level. According

to your comments and suggestions, we have made a very careful revision. The revisions are highlighted in

blue fonts in the revised version.

Your comment and suggestion: This paper presents a quantized intermittent sampled-data trans

mission protocol to solve the sampled-data exponential stabilization problem for delayed complex-valued

neural networks. The paper is original and technically interesting. However, it can be revised to further

improve its readability. Some discussions are not clearly stated which may hinder the readers’ understand

ing.

Our reply: Thanks the reviewer for having brought this valuable comments to our paper. We have

carefully addressed each point as detailed below.

Question 1: The description of the paper’s contributions in the introduction is vague.

Our reply: We thank the reviewer for this valuable comment. We have revised the introduction to

present the contributions of this paper more clearly and explicitly. Specifically, we have restructured the

contribution statement to highlight three distinct aspects:

(a) Different from existing quantized intermittent sampled-data transmission (QIST) schemes that are

only developed for real-valued neural networks, this work establishes a complete QIST control framework

for delayed complex-valued neural networks (DCNNs). A complex-domain state feedback controller is

designed, which significantly cuts network transmission frequency and actuator running time to improve

energy efficiency under limited communication bandwidth.

(b) A novel transmission-interval-dependent switched Lyapunov functional is specially constructed to

match the two-mode switching characteristic of the QIST protocol (transmission interval [tk,sk) and rest

interval [sk,tk+1)). Compared with conventional Lyapunov-Krasovskii functionals for intermittent control,

the proposed functional maintains continuity at switching instants and imposes much milder constraints

on Lyapunov matrices, which fully exploits sampled state information to reduce conservatism.

(c) Combining sector-bounded quantization characterization and advanced integral inequality techniques,

two less-conservative exponential stabilization criteria are derived in the form of complex-valued LMIs.

For the first time, our conditions quantitatively reveal the explicit coupling relationship among exponen

tial convergence rate, quantization density, maximum transmission length and rest-interval duty cycle.

A one-dimensional search algorithm is further provided to solve the admissible upper bound of the duty

cycle.

Question 2: The extensibility of the proposed method is not discussed. For instance, can this method

apply to large-scale systems, as considered in “decentralized adaptive delayed control of large-scale nonlin

ear systems with random couplings and biased measurements”?

Our reply: We thank the reviewer for pointing out this important missing discussion on method ex

tensibility and scalability to large-scale systems. We have incorporated this discussion as a newly added

Remark 5 at the end of Section 3 (Stability Analysis) in the revised manuscript.

Question 3: Why is the quantization scheme in (3) chosen? Can other types of quantizers also be

applied?

Our reply: We thank the reviewer for this question. We have clarified the motivation for choosing the

logarithmic quantizer in (3) by adding a new explanatory paragraph immediately after equation (3) in

Section 2.

The logarithmic quantizer in (3) is adopted for three main reasons. First, it admits a convenient sector

bound description q(v) − v = ∆(t)v with ||∆(t)|| ≤ δ, which enables the quantization error to be incor

porated into the subsequent LMI-based stability analysis in a tractable manner. Second, the quantization

density parameter ρ ∈ (0,1) provides a direct trade-off between quantization accuracy and communication

bit rate, which is crucial for capacity-limited channels. Third, this type of quantizer has been widely

validated in [45]–[50] and is standard for neural network systems with quantized feedback. It is worth

noting that other quantizer types, such as uniform quantizers, dynamic quantizers with adjustable scal

ing, or hysteresis quantizers, could also be considered in principle. However, uniform quantizers generally

lack the sector-bound property that facilitates LMI-based design, while dynamic and hysteresis quantizers

would introduce additional dynamics or switching logic that complicate the stability analysis. Extending

the proposed framework to accommodate these alternative quantization schemes is an interesting direction

for future investigation.

Question 4: The statement in Theorem 1 that the LMIs hold for arbitrary real matrices U, M, W,

and N seems questionable.

Our reply:We sincerely appreciate the reviewer for pointing out this critical wording error. The term

“arbitrary” was mistakenly used in the original manuscript, which leads to misleading interpretation.

Matrices U,M,W,N are auxiliary free-weighting matrices (decision variables) introduced in the stability

analysis via the free-weighting matrix technique. The correct mathematical meaning is that there exists

a set of suitable matrices U,M,W,N such that the LMIs are satisfied, rather than the LMIs hold for

all arbitrary choices of U,M,W,N. We have revised Theorem 1 in the revised manuscript accordingly,

removing the incorrect word “arbitrary” and rephrasing the description of these auxiliary matrices to avoid

confusion.

Question 5: The resolution of Fig. 1 is low.

Our reply: We have regenerated Fig. 1 from the original Visio source file. The figure is exported

as lossless 600 dpi TIFF file complying with PLOS ONE standards, eliminating blurriness caused by

low resolution. All other figures in the manuscript have also been rechecked and re-exported with high

resolution.

Thank you again for your constructive comments and your great help which improve the clarity of this

paper, and we hope the revised paper is satisfactory for publication.

Additional Revisions Addressing Journal Requirements

In addition to the above point-by-point responses, we have also addressed the following journal-specific

requirements:

Data Availability Statement: We have revised the Data Availability Statement to ensure full compliance

with PLOS ONE’s data policy. All data underlying the findings are fully available within the manuscript

and its supporting information.

Reference List Verification: We have carefully checked our reference list to ensure that it is complete,

correct, and does not contain any retracted articles. All cited references are appropriate and up to date.

ORCIDVerification: The corresponding author’s ORCID iD has been verified in the submission system.

We have also encouraged all coauthors to verify their ORCID iDs before final acceptance.

Language and Grammar: We have carefully proofread the entire manuscript to correct any typographical or grammatical errors.

Attachment Submitted filename: Response_to_Reviewers_auresp_2.pdf

# Peer review: Decision Letter 2

**DOI:** 10.1371/journal.pone.0357795.r005

**Authors:** Gang Wang

23 Aug 2026

Sampled-data exponential stabilization for delayed complex valued neural networks with quantized intermittent communication

PONE-D-26-02182R2

Dear Dr. wang,

We’re pleased to inform you that your manuscript has been judged scientifically suitable for publication and will be formally accepted for publication once it meets all outstanding technical requirements.

Within one week, you’ll receive an e-mail detailing the required amendments. When these have been addressed, you’ll receive a formal acceptance letter and your manuscript will be scheduled for publication.

An invoice will be generated when your article is formally accepted. Please note, if your institution has a publishing partnership with PLOS and your article meets the relevant criteria, all or part of your publication costs will be covered. Please make sure your user information is up-to-date by logging into Editorial Manager at Editorial Manager® and clicking the ‘Update My Information' link at the top of the page. For questions related to billing, please contact billing support.

If your institution or institutions have a press office, please notify them about your upcoming paper to help maximize its impact. If they’ll be preparing press materials, please inform our press team as soon as possible -- no later than 48 hours after receiving the formal acceptance. Your manuscript will remain under strict press embargo until 2 pm Eastern Time on the date of publication. For more information, please contact onepress@plos.org.

Kind regards,

Gang Wang

Academic Editor

PLOS One

Additional Editor Comments (optional):

Reviewers' comments:

Reviewer's Responses to Questions

**Comments to the Author**

Reviewer #3: All comments have been addressed

**********

2. Is the manuscript technically sound, and do the data support the conclusions?

Reviewer #3: Yes

**********

3. Has the statistical analysis been performed appropriately and rigorously?

Reviewer #3: Yes

**********

4. Have the authors made all data underlying the findings in their manuscript fully available?

The PLOS Data policy

Reviewer #3: No

**********

5. Is the manuscript presented in an intelligible fashion and written in standard English?

Reviewer #3: Yes

**********

Reviewer #3: I am satisfied with the revisions made in light of the comments. Therefore, I have no further concerns.

**********

what does this mean?). If published, this will include your full peer review and any attached files.

If you choose “no”, your identity will remain anonymous but your review may still be made public.

**Do you want your identity to be public for this peer review?**  For information about this choice, including consent withdrawal, please see our Privacy Policy

Reviewer #3: No

**********

# Editor report: Acceptance letter

**DOI:** 10.1371/journal.pone.0357795.r006

**Authors:** Gang Wang

PONE-D-26-02182R2

PLOS One

Dear Dr. Wang,

I'm pleased to inform you that your manuscript has been deemed suitable for publication in PLOS One. Congratulations! Your manuscript is now being handed over to our production team.

At this stage, our production department will prepare your paper for publication. This includes ensuring the following:

* All references, tables, and figures are properly cited

* All relevant supporting information is included in the manuscript submission,

* There are no issues that prevent the paper from being properly typeset

You will receive further instructions from the production team, including instructions on how to review your proof when it is ready. Please keep in mind that we are working through a large volume of accepted articles, so please give us a few days to review your paper and let you know the next and final steps.

Lastly, if your institution or institutions have a press office, please let them know about your upcoming paper now to help maximize its impact. If they'll be preparing press materials, please inform our press team within the next 48 hours. Your manuscript will remain under strict press embargo until 2 pm Eastern Time on the date of publication. For more information, please contact onepress@plos.org.

You will receive an invoice from PLOS for your publication fee after your manuscript has reached the completed accept phase. If you receive an email requesting payment before acceptance or for any other service, this may be a phishing scheme. Learn how to identify phishing emails and protect your accounts at https://explore.plos.org/phishing.

If we can help with anything else, please email us at customercare@plos.org.

Thank you for submitting your work to PLOS One and supporting open access.

Kind regards,

PLOS One Editorial Office Staff

on behalf of

Dr. Gang Wang

Academic Editor

PLOS One
